# Delivery of an immunogenic cell death-inducing copper complex to cancer stem cells using polymeric nanoparticles†

**DOI:** 10.1039/d1ra08788f

**Published:** 2022-02-11

**Authors:** Ginevra Passeri, Joshua Northcote-Smith, Kogularamanan Suntharalingam

**Affiliations:** School of Chemistry, University of Leicester Leicester LE1 7RH UK k.suntharalingam@leicester.ac.uk

## Abstract

The major cause for cancer related deaths worldwide is tumour relapse and metastasis, both of which have been heavily linked to the existence of cancer stem cells (CSCs). CSCs are able to escape current treatment regimens, reform tumours, and promote their spread to secondary sites. Recently, our research group reported the first metal-based agent 1 (a copper(ii) compound ligated by a bidentate 4,7-diphenyl-1,10-phenanthroline and a tridentate Schiff base ligand) to potently kill CSCs *via* cytotoxic and immunogenic mechanisms. Here we show that encapsulation of 1 by polymeric nanoparticles at the appropriate feed (10%, 1 NP^10^) enhances CSC uptake and improves potency towards bulk cancer cells and CSCs (grown in monolayer and three-dimensional cultures). The nanoparticle formulation triggers a similar cellular response to the payload, which bodes well for further translation. Specifically, the nanoparticle formulation elevates intracellular reactive oxygen species levels, induces ER stress, and evokes damage-associated molecular patterns consistent with immunogenic cell death. To the best of our knowledge, this is the first study to demonstrate that polymeric nanoparticles can be used to effectively deliver immunogenic metal complexes into CSCs.

## Abstract 2

In this study we deliver an immunogenic cell death-inducing copper(ii) complex, comprising of 4,7-diphenyl-1,10-phenanthroline and a Schiff base ligand, to breast cancer stem cells.

## Graphical abstract

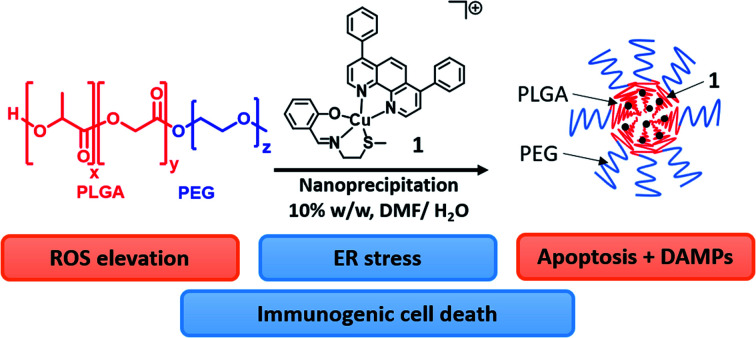

## Introduction

Cancer stem cells (CSCs) are a small sub-population of tumours with the capacity to differentiate, self-renew, and form secondary tumours.^1,2^ CSCs can evade current chemotherapeutic and radiotherapeutic approaches as these treatments tend to target fast growing cancer cells, and CSCs, due to their quiescent stem cell-like nature, divide relatively slowly.^3–5^ As CSCs only make up a small fraction of any given tumour and reside in hard to reach niches, they are often missed by surgical interventions as well.^6^ After escaping conventional treatment regimens, CSCs can reform tumour mass within the original site or promote spread and tumour anchorage at secondary sites (metastasis).^7,8^ Considering the negative clinical implications of CSCs, it is imperative that cancer therapies have the ability to remove heterogeneous tumour populations in their entirety, including CSCs. Thus far, an anti-CSC agent that is effective in clinical settings has not been reported, this is despite the identification of targetable CSC features such as constituents of the CSC microenvironment, overexpressed plasmatic membrane proteins, and overactive cell signalling pathways.^9–11^ The use of external chemical agents or biologics to prompt the immune system to target and remove tumours is now emerging as a viable oncological approach, that could provide effective, long-term outcomes.^12^ Contemporary research in the cancer immunology field suggests that certain cancer treatments can be improved by incorporating CSC-targeting immunotherapeutic agents within regimens that solely rely on cytotoxic drugs.^13,14^ Therefore the design and development of immuno-chemotherapeutic agents that can kill CSCs *via* immunogenic and cytotoxic mechanisms could vastly improve the repertoire of treatment options available to oncologists to treat cancer patients (particularly those suffering from or prone to relapse and metastasis).

**Fig. 1:**
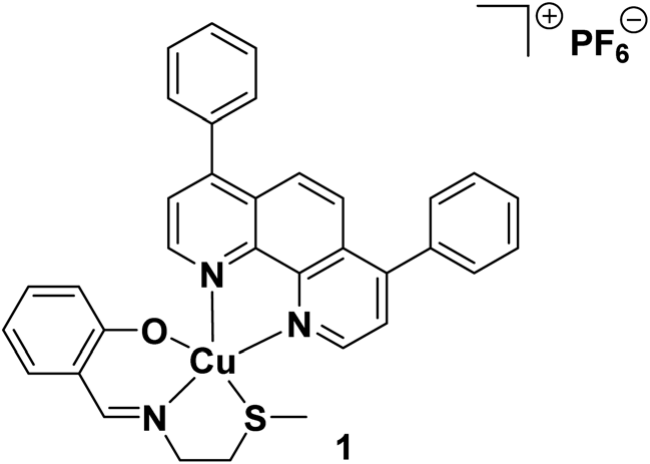
Chemical structure of a copper(ii) complex 1 capable of inducing immunogenic cell death of breast CSCs.

Exogenous agents can impart a cancer cell-targeting immune response by evoking an atypical mode of cell death called immunogenic cell death (ICD), whereby non-viable cancer cells prompt immune cells within the tumour microenvironment to find, envelop, process, and destroy them by revealing specific protein signals.^15^ Applying the same school of thought to CSCs, it is reasonable to envisage that CSCs that have endured ICD have the potential to act as so-called ‘vaccines’ and prompt an adaptive immune response against other CSCs with similar chemical compositions. The capacity of chemical agents to trigger ICD of cancer cells is directly linked to their ability to localise in the endoplasmic reticulum (ER) and elevated reactive oxygen species (ROS) levels, which often leads to ER stress and apoptosis.^16,17^ Such ER-targeting, ROS-generating, ICD-inducing chemical agents are known as Type II ICD inducers. There are very few genuine Type II ICD inducers reported to date, and of these examples, few have been shown to target CSCs of any tissue type and only a handful contain a metal.^18,19^ Very recently, our research group reported a copper(ii)-containing compound 1, made up of a bidentate 4,7-diphenyl-1,10-phenanthroline ligand and a tridentate Schiff base ligand, that was able to induce ICD of CSCs (see Fig. 1 for chemical structure of 1).^20^ The copper(ii) complex 1 was the first inorganic compound to kill CSCs (of any tissue type) in an immunogenic manner. This discover was a positive step toward the development of clinically applicable metal-based immuno-chemotherapeutics, as the removal of CSCs by immunogenic agents in tandem with traditional bulk cancer cell-active treatments, could prove to be an effective way of removing heterogeneous tumour populations in their entirety. Although the copper(ii) agent 1 displayed very promising anti-CSC properties *in vitro* further translation was curtailed due to its limited stability in physiologically relevant solutions (see Results and discussion section for detailed discussion). This shortcoming can be addressed by employing an appropriate delivery system that can effectively encapsulate the copper(ii) agent 1, thus providing protection against degradation prior to deliver into CSCs.

Nano-sized drug delivery systems offer a strategy to deliver drugs (including metal-based chemotherapeutics) to specific regions within the body (such as tumour microenvironments).^21^ Nanoparticles also offer distinct advantages over the free payload with respect to pharmacokinetics, including but not limited to improved drug solubility, higher bioavailability, and extended half-life.^22,23^ Nanoparticles can accumulate in certain tumours by taking advantage of the enhanced permeability and retention (EPR) effect.^24,25^ A chemical diverse range of nanoparticle formulations have been used for drug delivery, such as those constructed with iron oxide, carbon, gold, hydrogels, liposomes, and polymers.^26^ Some of these formulations are currently used in the clinic to deliver chemotherapeutics to tumours.^27^ Polymeric nanoparticles are of particular interest from a chemical point-of-view due to their biocompatibility, synthetic versatility, and tuneable properties.^28^ We recently reported two proof-of-concept studies where methoxy poly (ethylene glycol)-*b*-poly(d,l-lactic-*co*-glycolic) acid (PEG–PLGA), a biodegradable amphiphilic copolymer, was used to encapsulate and deliver copper(ii)- and manganese(ii)-nonsteroidal anti-inflammatory drug (NSAID) complexes into CSCs.^29,30^ The nanoparticle formulations improved both cellular uptake and cytotoxicity toward CSCs relative to the respective payloads.^29,30^ Here we use a similar polymeric nanoparticle formulation to encapsulate and deliver the ICD-inducing copper(ii) agent 1 into CSCs. Polymeric nanoparticles have been previously used to deliver cytotoxic agents into CSCs, however, this is as far as we are aware, the first study to demonstrate that polymeric nanoparticles can be used to effectively deliver an immunogenic metal complex into CSCs.

## Results and discussion

### Solution stability of the ICD-inducing copper(ii) agent 1

The solution stability of the ICD-inducing copper(ii) agent 1 was evaluated using biophysical techniques, namely UV-Vis spectroscopy and ESI mass spectrometry. According to UV-Vis spectroscopy studies, the absorbance trace of 1 (25 μM) was largely unchanged over the course of 24 h at 37 °C, in DMSO, DMF, or PBS : DMSO (200 : 1) with 10% FBS (Fig. S1–S3†). This is indicative of stability under these conditions. In contrast, the absorbance trace of 1 (25 μM) changed significantly over the course of 24 h at 37 °C, in PBS : DMSO (200 : 1) in the presence of ascorbic acid or glutathione (10 equivalents, well known cellular reductants) (Fig. S4 and S5†). This is indicative of instability under biologically reducing conditions. Further UV-Vis spectroscopy studies showed that when 1 (50 μM) in PBS : DMSO (200 : 1) in the presence of ascorbic acid or glutathione (10 equivalents) was subject to bathocuproine disulfonate (BCS, 2 equivalents), a strong copper(i) chelator, a characteristic absorbance band at 480 nm corresponding to [Cu^I^(BCS)_2_]^3−^ was observed (Fig. S6 and S7†). This implies that the copper(ii) centre in 1 undergoes reduction to the copper(i) form in the presence of ascorbic acid or glutathione.^31^ ESI mass spectrometry studies revealed that upon incubation of 1 (500 μM) in H_2_O : DMSO (10 : 1) with ascorbic acid or glutathione (10 equivalents), [Cu^I^(4,7-diphenyl-1,10-phenanthroline)_2_]^+^ was produced (Fig. S8†). The ESI (positive) mass spectrum of the solution displayed a dominant molecular ion peak corresponding to [Cu^I^(4,7-diphenyl-1,10-phenanthroline)_2_]^+^ (727 *m*/*z*) with the appropriate isotopic pattern, and no molecular ion peak corresponding to unmodified 1 (Fig. S8†). Collectively this suggests that in the presence of bioreductants, the copper(ii) centre in 1 is prone to undergo reduction to copper(i), which in turn promotes ligand exchange (displacement of a Schiff base ligand with a 4,7-diphenyl-1,10-phenanthroline ligand in this case). The structural reorganisation of 1 to [Cu^I^(4,7-diphenyl-1,10-phenanthroline)_2_]^+^ is consistent with the geometrical preferences of copper(ii) and copper(i) compounds. The copper(ii) complex 1 adopts a distorted trigonal bipyramidal geometry which is consistent with a copper(ii), d^9^ centre whereas the reduced analogue is likely to adopt a distorted tetrahedral geometry consistent with a copper(i), d^10^ centre. Although the ESI mass spectrometry studies identified the reduced form of 1 to be [Cu^I^(4,7-diphenyl-1,10-phenanthroline)_2_]^+^, this is unlikely to be the major reduced product *in vivo*. Biological systems contain large pools of nucleophiles with high copper(i) affinities and soft donor sites. These biological nucleophiles will undoubtedly outcompete the 4,7-diphenyl-1,10-phenanthroline and Schiff base ligands for the soft copper(i) centre to form copper(i)-biomolecule complexes. Overall, the UV-Vis spectroscopy and ESI mass spectrometry studies show that although 1 is stable in organic solvents such as DMSO and DMF, and physiologically relevant solutions such as PBS with 10% FBS, the copper(ii) centre in 1 is susceptible to reduction under biologically reducing conditions, which promotes undesirable structural transformations. Therefore, in order to deliver 1 into CSCs (within a biological system), in its unmodified form, a suitable drug delivery system is needed.

### Encapsulation of the ICD-inducing copper(ii) agent 1 into polymeric nanoparticles

**Fig. 2:**
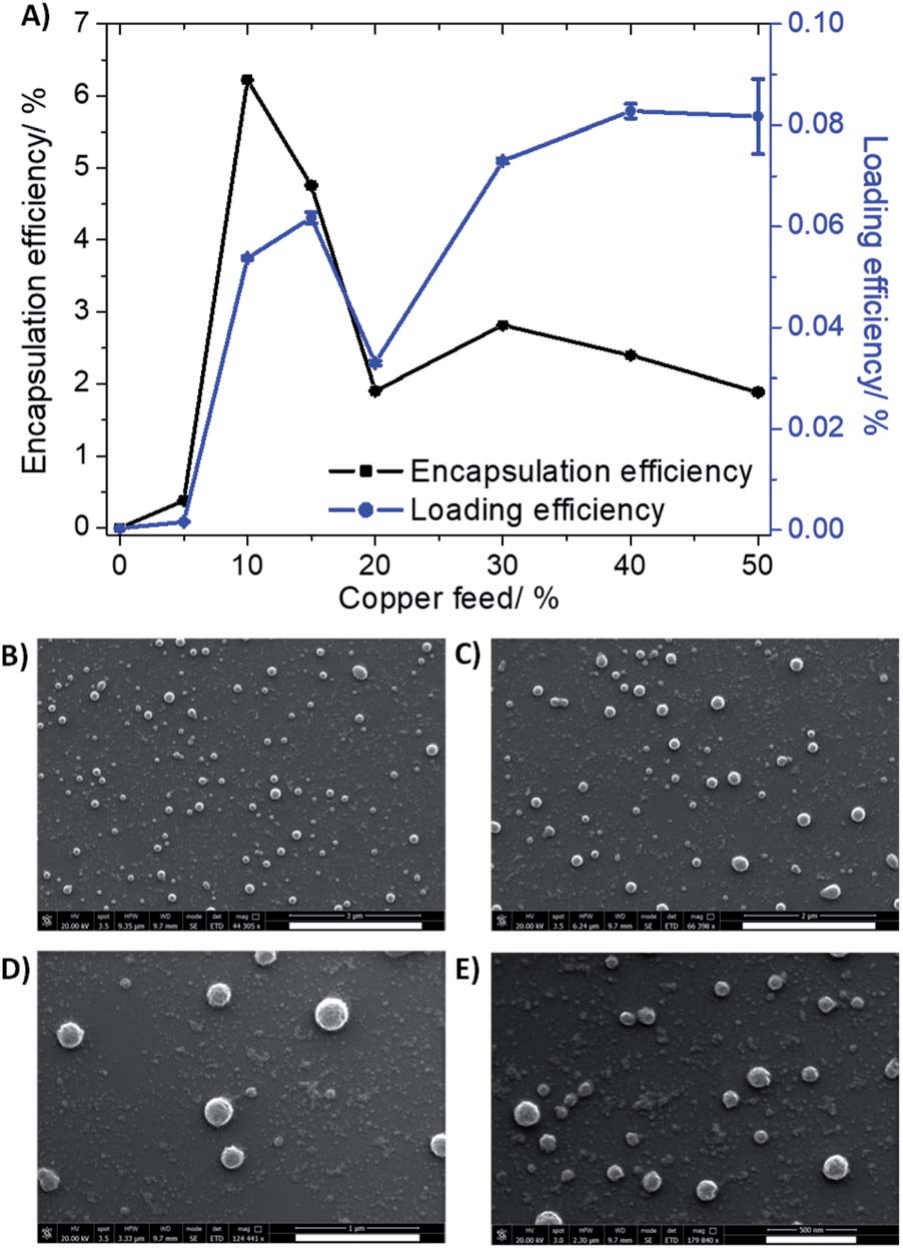
(A) The effect of feed variation on loading and encapsulation efficiency of 1 incorporated into PEG–PLGA (5000 : 20 000 Da, 1 : 1 LA : GA) nanoparticles. Scanning electron microscopy (SEM) images of 1 NP^10^ suspended in water at (B) ×44.3 magnification, scale bar = 3 μm, (C) ×66.4 magnification, scale bar = 2 μm, (D) ×124.4 magnification, scale bar = 1 μm, and (E) ×179.8 magnification, scale bar = 500 nm.

To improve the translatable scope of the ICD-inducing copper(ii) agent 1, biodegradable and biocompatible PEG–PLGA polymeric nanoparticles were used. PEG–PLGA copolymers are amphiphilic. Therefore, when PEG–PLGA polymers (with the appropriate molecular weight) are added to aqueous solutions, they tend to self-assemble to form spherical nano-sized particles with a hydrophilic exterior made up of PEG and a hydrophobic interior comprising of PLGA. As the ICD-inducing copper(ii) agent 1 is relatively hydrophobic (Log *P* = 2.01 ± 0.16), the nanoprecipitation method was employed to encapsulate 1 into the hydrophobic core of PEG–PLGA (5000 : 20 000 Da, 1 : 1 LA : GA) nanoparticles. Various nanoparticle formulations of 1 and PEG–PLGA were prepared (1 NP^5–50^) by altering the feed (percentage of 1 to PEG–PLGA polymer in terms of mass) between 5% and 50%. The amount of copper in each formulation was determined by inductively coupled plasma mass spectrometry (ICP-MS) after digestion by concentrated nitric acid, and this was used to calculate the loading and encapsulation efficiency of 1, and determine the most appropriate formulation for *in vitro* evaluation. As depicted in Fig. 2A, the calculated loading and encapsulation efficiency of 1 varied with feed. Based on the data acquired, the optimal encapsulation conditions were achieved at 10% feed (1 NP^10^). At 10% feed (1 NP^10^) the encapsulation efficiency was 6.22 ± 0.003% and the loading efficiency was 0.05 ± 0.0003%.

Characterisation of 1 NP^10^ by dynamic light scattering (DLS) revealed that the nanoparticle diameter was 108.7 ± 0.8 nm, and the polydispersity was 0.112 ± 0.006 (Fig. S9†). The diameter of 1 NP^10^ was 17% higher than the corresponding empty PEG–PLGA nanoparticle (92.8 ± 1.9 nm, Fig. S10†) indicative of encapsulation of 1 into the lipophilic core of the PEG–PLGA nanoparticle. Furthermore, the diameter and polydispersity of 1 NP^10^ was consistent with previously reported metal complex–polymer nanoparticle formulations.^29,30,32^ The surface morphology and size distribution of 1 NP^10^ was assessed by scanning electron microscopy (SEM). The SEM images confirmed that 1 NP^10^ adopted relatively uniform spherical structures with an average size of 118.3 ± 9.6 nm (Fig. 2B). The average nanoparticle size determined using SEM analysis is in good agreement with the DLS measurements. The solution stability of 1 NP^10^ was gauged by monitoring its size over the course of 72 h in physiologically relevant buffered solutions. The size of 1 NP^10^ was largely unaltered in water, PBS with 10% FBS, and mammary epithelial growth medium (MEGM) (all at pH 7.4) over the course of 72 h at 37 °C (Fig. S11†), indicative of reasonable stability. The solution stability of 1 NP^10^ bodes well for the potential delivery of 1 into CSCs, in its intact form.

### Delivery of 1 into CSCs using polymeric nanoparticles

ICP-MS was used to determine the ability of the nanoparticle formulation 1 NP^10^ to internalise the payload 1 into bulk breast cancer cells (HMLER) and breast CSCs (HMLER-shEcad). Specifically, HMLER and HMLER-shEcad cells were independently treated with 1 NP^10^ (110 nM) for 24 h at 37 °C, harvested, digested, and analysed for copper. A relatively large amount of the nanoparticle formulation 1 NP^10^ was internalised by both HMLER and HMLER-shEcad cells. As depicted in Fig. S12,† 63.4 ± 1.8 ng of Cu per million cells was detected in 1 NP^10^-treated HMLER cells and 93.2 ± 1.3 ng of Cu per million cells was detected in 1 NP^10^-treated HMLER-shEcad cells. Identical uptake studies were also carried out with the payload 1 (110 nM for 24 h at 37 °C). The intracellular copper concentration of 1-treated HMLER cells and 1-treated HMLER-shEcad cells was 9.4- to 12.4-fold lower (6.7 ± 0.1 to 7.5 ± 0.1 ng of Cu per million cells, Fig. S12†) than that detected for 1 NP^10^-treated cells. This result clearly shows that the encapsulation of 1 into PEG–PLGA nanoparticles improves its ability to be taken up by both bulk breast cancer cells and breast CSCs.

To identify if the uptake of 1 NP^10^ by HMLER and HMLER-shEcad cells was active or passive, temperature dependent cellular uptake experiments were conducted. Specifically, HMLER and HMLER-shEcad cells were treated with 1 NP^10^ (110 nM for 4 h) at 4 °C and 37 °C and the copper content in the respective cells was measured by ICP-MS (Fig. S13†). HMLER and HMLER-shEcad cells treated with 1 NP^10^ at 4 °C displayed a 71% and 89% decrease in copper uptake, respectively, compared to the same cells treated with 1 NP^10^ at 37 °C. The temperature-dependent uptake observed for 1 NP^10^ is suggestive of an active process. Nanoparticles made up of PEG–PLGA polymers are well known to be internalised by cells *via* endocytosis.^33^ To determine if 1 NP^10^ is taken up by HMLER-shEcad cells *via* endocytosis, cellular uptake studies were performed in the presence of endocytosis inhibitors. More specifically, HMLER-shEcad cells were pre-treated with endocytosis inhibitors, ammonium chloride (50 mM for 2 h) and chloroquine (100 μM for 2 h) and then treated with 1 NP^10^ (16 nM for 24 h at 37 °C), after which the cells were harvested, digested, and analysed for copper by ICP-MS. As expected a significant decrease (*p* < 0.05) in 1 NP^10^ uptake was observed in the presence of the inhibitors, indicating that 1 NP^10^ does indeed enter breast CSC-enriched HMLER-shEcad cells *via* an endocytic mechanism (Fig. S14†). Macromolecular agents, including nanoparticles, internalised by cells *via* endocytosis enter the cytoplasm through endosomes. Endosomes are a collection of intracellular sorting organelles with acidic vesicles. Given that 1 NP^10^ is most likely taken up into cells *via* endosomes, the ability of 1 NP^10^ to release its payload 1 under conditions resembling acidic endosomal vesicles (sodium acetate buffer, pH 5.2 at 37 °C) was determined. The nanoparticle formulation 1 NP^10^ released 80% of its payload under these conditions over 72 h (Fig. S15†). In physiologically neutral conditions (PBS, pH 7.4 at 37 °C), 1 NP^10^ was only able to release 29% of its payload over 72 h (Fig. S15†). Taken together this implies that 1 NP^10^ is capable of selectively releasing 1 in acidic compartments within cells (such as endosomes) upon endocytic uptake.

### Bulk breast cancer and breast CSC toxicity of the nanoparticle formation 1 NP^10^ in monolayer and three-dimensional cultures

The potency of the nanoparticle formulation 1 NP^10^ towards breast CSC-enriched (HMLER-shEcad) and breast CSC-depleted (HMLER) cells, cultured in monolayer systems, was determined using the MTT assay. The IC_50_ values (concentration required to reduce cell viability by 50%) were determined from dose–response curves (Fig. S16†) and are summarised in Table 1. The nanoparticle formulation displayed nanomolar toxicity towards both HMLER and HMLER-shEcad cells. The nanoparticle formulation 1 NP^10^ was 7- to 16-fold more toxic than the payload 1 for HMLER and HMLER-shEcad cells.^20^ The differential toxicities could be related to the significantly better internalisation of 1 when administered as the nanoparticle formulation as opposed to the free metal complex. Importantly, 1 NP^10^ killed HMLER and HMLER-shEcad cells with similar potency (akin to 1), showing that the encapsulation of 1 by PEG–PLGA polymeric nanoparticles does not affect its spectrum of activity. Notably, the potency of 1 NP^10^ for CSC-enriched HMLER-shEcad cells was 210-fold greater than that of salinomycin, an established clinically-tested anti-breast CSC agent.^34^ The empty PEG–PLGA nanoparticle was non-toxic towards both HMLER and HMLER-shEcad cells (IC_50_ value > 100 μM) (Fig. S17†). This implies that the potency of the nanoparticle formation 1 NP^10^ is wholly due to its payload 1, and there is little or no contribution from the PEG–PLGA carrier.

**Table 1:** IC_50_ values of the nanoparticle formulation 1 NP^10^, the payload 1, the empty PEG–PLGA nanoparticle, and salinomycin against HMLER and HMLER-shEcad cells and HMLER-shEcad mammospheres determined after 72 h or 120 h incubation (mean of three independent experiments ± SD)

Compound	HMLER IC_50_/μM	HMLER-shEcad IC_50_/μM	Mammosphere IC_50_/μM
1 NP^10^	0.03 ± 0.002	0.02 ± 0.004	0.10 ± 0.01
1^a^	0.21 ± 0.01	0.32 ± 0.02	0.54 ± 0.01
Empty NP	>100	>100	>33
Salinomycin^b^	11.40 ± 0.40	4.20 ± 0.30	18.50 ± 1.50

a Taken from ref. 20.

b Taken from ref. 34 and 35.

**Fig. 3:**
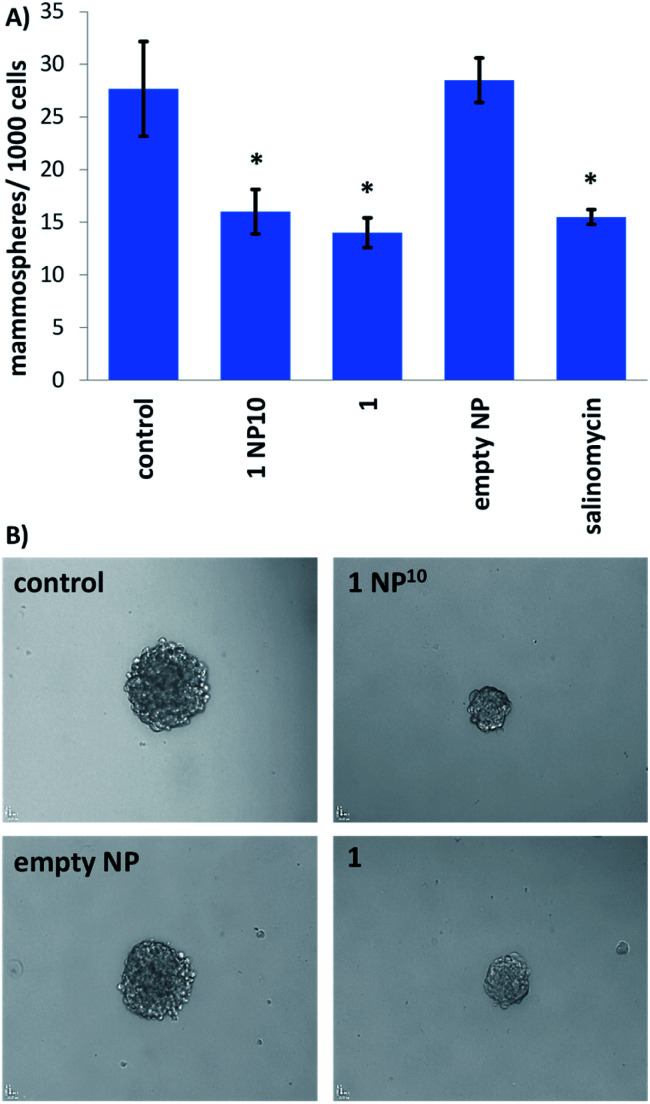
(A) Bar chart representing the number of mammopsheres formed per thousand HMLER-shEcad cells in single cell suspensions in the absence and presence of 1 NP^10^, 1, the empty PEG–PLGA nanoparticle, or salinomycin (at their respective IC_20_ values) after 5 days incubation. Error bars = SD and Student's *t*-test, * = *p* < 0.05. (B) Bright-field images (×10) of mammospheres formed from HMLER-shEcad cells in single cell suspensions untreated and treated with 1 NP^10^, 1, or the empty PEG–PLGA nanoparticle (at their respective IC_20_ values for 5 days).

Breast CSCs when cultured under low-attachment conditions with no serum supplements form multicellular structures called mammospheres. Mammospheres are collections of free-floating breast CSCs arranged in three-dimensional spheroids. As three-dimensional cultures are more representative of organs and tumours than monolayer cell cultures, the ability of a given agent to inhibit mammosphere formation with respect to number, size, and viability, serves as a useful gauge of its *in vivo* potential. Mammosphere formation studies showed that single cell suspensions of HMLER-shEcad cells, when treated with 1 NP^10^ (at the IC_20_ value for 5 days), were significantly less able to form mammospheres than untreated cells (Fig. 3). The payload 1 and salinomycin had a similar effect to 1 NP^10^ on mammosphere formation (when treated at their respective IC_20_ values for 5 days) (Fig. 3 and S18†). This shows that encapsulation of 1 into PEG–PLGA polymeric nanoparticles does not detrimentally effect its mammosphere inhibitory properties. As expected, the empty PEG–PLGA nanoparticle did not significantly affect the number and size of HMLER-shEcad mammospheres formed (Fig. 3). To determine the ability of 1 NP^10^ to reduce mammosphere viability, TOX8 a resazurin-based reagent was used. The IC_50_ values (concentration required to reduce mammosphere viability by 50%) were interpolated from dose–response curves (Fig. S19†) and are summarised in Table 1. The nanoparticle formulation 1 NP^10^ was 5.4-fold more potent towards HMLER-shEcad mammospheres than the payload 1, indicating that encapsulation of 1 into PEG–PLGA polymeric nanoparticles improves its mammosphere potency.^20^ Also, 1 NP^10^ was significantly (185-fold, *p* < 0.05, *n* = 6) more toxic towards HMLER-shEcad mammospheres than salinomycin.^35^ The empty PEG–PLGA nanoparticle was relatively non-toxic towards HMLER-shEcad mammospheres (IC_50_ value > 33 μM) (Fig. S20†). In light of the monolayer and three-dimensional toxicity data, it is evident that the potency of 1 towards breast CSCs (HMLER-shEcad cells) is significantly enhanced by encapsulation into PEG–PLGA polymeric nanoparticles (1 NP^10^).

### Mechanism of action of the nanoparticle formation 1 NP^10^

**Fig. 4:**
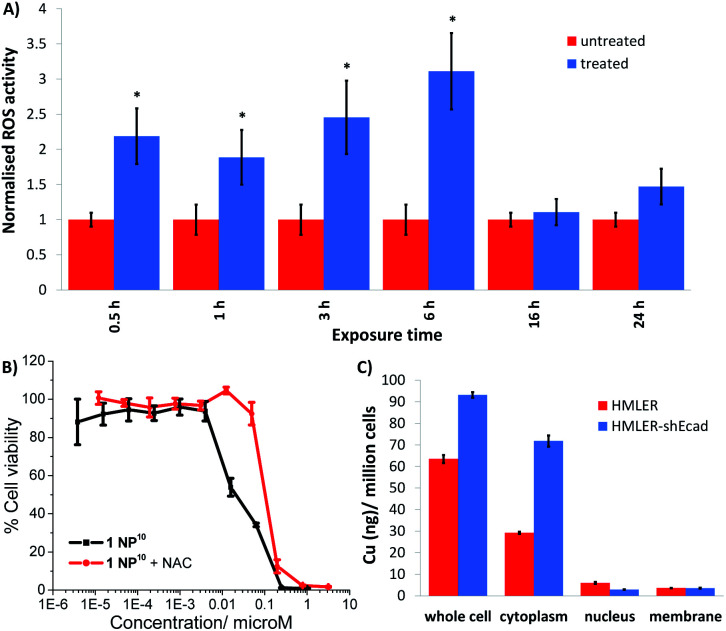
(A) Bar chart representing the relative intracellular ROS levels in HMLER-shEcad cells in the absence (control) and presence of 1 NP^10^ (IC_50_ value) at various time-points over the course of 24 h. DCFDA was used to detect intracellular ROS levels. Error bars = SD and Student's *t*-test, * = *p* < 0.05. (B) Dose–response curves depicting the effect of 1 NP^10^ on HMLER-shEcad cell viability with and without the ROS scavenger, *N*-acetylcysteine (2.5 mM) after 72 incubation. (C) Bar chart representing the amount of copper detected by ICP-MS in the whole cell, cytoplasm, nucleus, and membrane fractions isolated from HMLER and HMLER-shEcad cells incubated with 1 NP^10^ (110 nM) for 24 h.

Further cell-based studies were carried out to determine the mechanism of action of the nanoparticle formulation 1 NP^10^, and to determine whether it was consistent with the payload 1. Given that the payload 1 was previously shown to induce ROS-mediated ER stress and subsequent ICD of breast CSCs,^20^ we initially probed the ability of 1 NP^10^ to elevate intracellular ROS levels. The ability of 1 NP^10^ to increase intracellular ROS levels was monitored using 2′,7′-dichlorodihydrofluorescein diacetate (DCFDA). DCFDA is a fluorescent dye that is widely used to accurately measure intracellular ROS levels. A time-dependent increase in intracellular ROS levels was observed in HMLER-shEcad cells treated with 1 NP^10^ (at the IC_50_ value) up to 6 h exposure (1.9- to 3.1-fold increase in detectable ROS levels compared to untreated cells, *p* < 0.05; Fig. 4A). Prolonged exposure (16 and 24 h) did not increase intracellular ROS levels to the same extent as shorter exposure (Fig. 4A). Similar time-dependent ROS production was previously reported for 1, however the maximal ROS production (relatively to untreated control cells) was significantly higher for 1 NP^10^-treated HMLER-shEcad cells (311% increase after 6 h exposure) than 1-treated HMLER-shEcad cells (43% increase after 16 h exposure).^20^ The enhanced ROS generation by 1 NP^10^ compared to 1 could be related to the greater internalisation of the nanoparticle formulation compared to the free payload at the administered concentration. Independent cytotoxicity studies showed that the potency of 1 NP^10^ towards HMLER-shEcad cells decreased significantly (IC_50_ value increased 5-fold to 0.10 ± 0.003 μM, *p* < 0.05, *n* = 18) when co-incubated with *N*-acetylcysteine (NAC), a ROS scavenger (2.5 mM, 72 h) (Fig. 4B). This further proves that 1 NP^10^-induced breast CSC death is related to intracellular ROS perturbations. A comparable outcome in terms of attenuation of potency in the presence of NAC was previously observed for the payload 1.^20^ This demonstrates that the encapsulation of 1 into PEG–PLGA polymeric nanoparticles does not diminish its ability to kill breast CSC *via* a ROS-dependent mechanism.

Cellular fractionation studies were performed to determine the intracellular distribution of 1 NP^10^ in bulk breast cancer cells and breast CSCs. HMLER and HMLER-shEcad were treated with 1 NP^10^ (110 nM at 37 °C for 24 h) and the cytoplasmic, nuclear, membrane fractions were extracted and the copper content was measured by ICP-MS (Fig. 4C). This revealed that 1 NP^10^ predominantly accumulated in the cytoplasm, at levels 4.8- to 24.2-fold and 8.1- to 20.0-fold higher than in the nucleus and membrane respectively. This finding and the fact that the payload 1 induces ER stress, led us to investigate the possibility that 1 NP^10^ may also induce ER stress and activate the unfolded protein response (UPR). Co-administration of 1 NP^10^ and salubrinal (10 μM), an inhibitor of eIF2α phosphatase that works synergistically with ER stress inducers to enhance their potency,^36^ significantly increased the cytotoxicity of 1 NP^10^ towards HMLER-shEcad cells (IC_50_ value decreased from 0.02 ± 0.004 μM to 0.008 ± 0.0003 μM, *p* < 0.05, *n* = 18; Fig. S21†). This suggests that ER stress is a component of the cytotoxic mechanism of 1 NP^10^. To further prove ER stress, we monitored the expression of proteins related to the UPR in 1 NP^10^-treated breast CSCs.^37^ HMLER-shEcad cells treated with 1 NP^10^ (40–160 nM for 4 h) displayed a noticeable increase in the expression of phosphorylated eukaryotic initiation factor 2α (phos-eIF2α) while unphosphorylated eIF2α levels remained largely unaltered (Fig. S22†), indicative of UPR activation. Phos-eIF2α promotes selective translation of the stress-related activating transcription factor-4 (ATF-4), which in turn can instigate apoptosis by upregulating C/EBP homologous protein (CHOP) expression.^38,39^ Activating transcription factor-6 (ATF-6), once cleaved can translocate to the nucleus and akin to ATF-4, activate transcription of CHOP, as well as ER chaperones.^40–42^ HMLER-shEcad cells incubated with 1 NP^10^ (40–160 nM for 4 h), displayed higher levels of ATF-4 and lower levels of ATF-6 compared to untreated cells (Fig. S22†), further proving UPR stimulation. CHOP was also markedly upregulated in HMLER-shEcad cells treated with 1 NP^10^ (95–191 nM for 24 h) (Fig. S22†). ER stress, if left unchecked, can lead to apoptosis.^43^ HMLER-shEcad cells treated with 1 NP^10^ (37–146 nM for 72 h) displayed higher levels of cleaved caspase 3 and 7, and poly ADP ribose polymerase (PARP) than untreated cells (Fig. S23†), characteristic of caspase-dependent apoptosis. Independent cytotoxicity studies showed that the potency of 1 NP^10^ towards HMLER-shEcad cells significantly decreased when co-administered with z-VAD-FMK (5 μM), a peptide-based caspase inhibitor (IC_50_ value increased from 0.02 ± 0.004 μM to 0.07 ± 0.001 μM, *p* < 0.05, *n* = 18; Fig. S24†). This suggests that 1 NP^10^ induces caspase-dependent apoptosis of breast CSCs. Collectively the immunoblotting and cytotoxicity studies indicate that 1 NP^10^, alike the payload 1, can induce ER stress and subsequent apoptotic breast CSC death.^20^

**Fig. 5:**
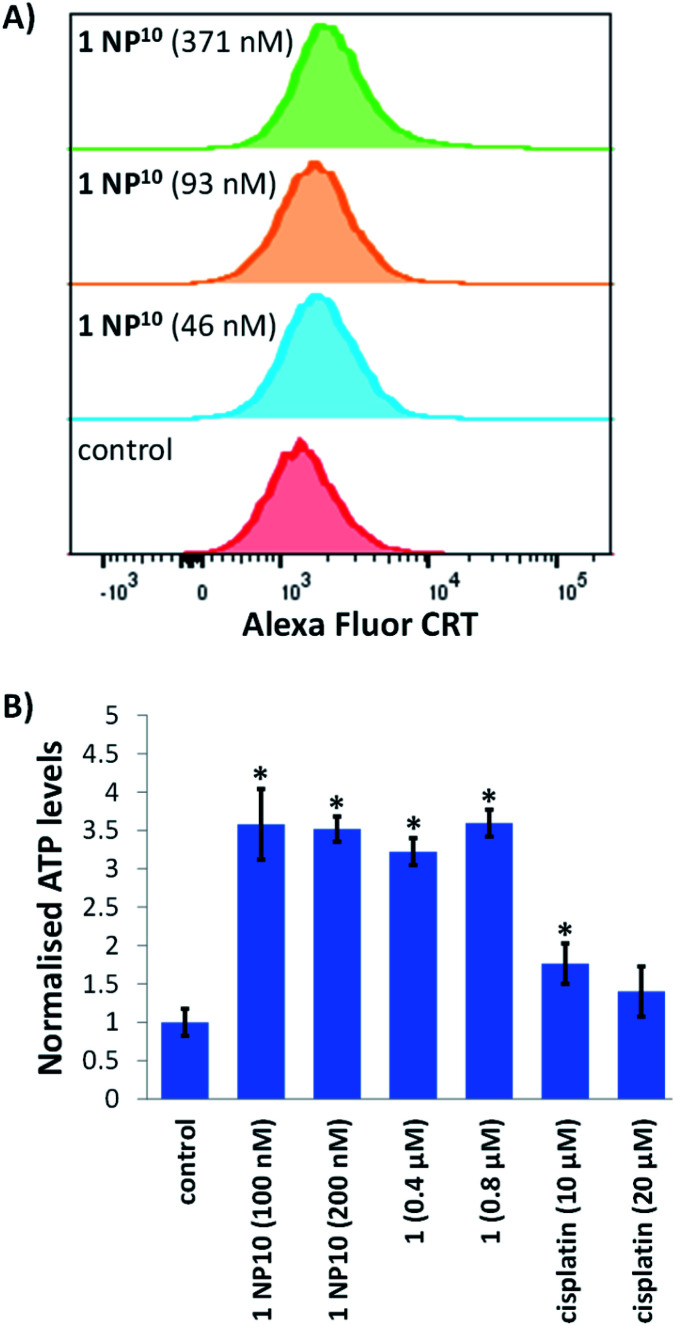
(A) Representative histograms displaying the green fluorescence emitted by anti-CRT Alexa Fluor 488 nm antibody-stained HMLER-shEcad cells untreated (red), and treated with 1 NP^10^ (46 nM for 24 h, 93 nM for 24 h, or 371 nM for 24 h) (blue, orange, and green). (B) Normalised extracellular ATP released from HMLER-shEcad cells untreated and treated with 1 NP^10^ (100–200 nM for 24 h), 1 (0.4–0.8 μM for 24 h), or cisplatin (10–20 μM for 24 h). Error bars = SD and Student's *t*-test, * = *p* < 0.05.

Next, we explored the ability of the nanoparticle formulation 1 NP^10^ to induce ICD of breast CSCs. There are three well-characterised hallmarks of ICD: the extracellular release of ATP and high mobility group box-1 (HMGB-1), and the translocation of calreticulin (CRT) from the ER to the plasmatic membrane.^17^ These hallmark are referred to as damage associated molecular patterns (DAMPs) and are vital for the recognition of death cells by immune cells and their consequential phagocytic engulfment. CRT exposed on the cell membrane of dying cells acts as an “eat me” signal, which promotes phagocytosis.^44,45^ The translocation of CRT to the plasma membrane was assessed using flow cytometry. HMLER-shEcad cells treated with 1 NP^10^ (46–371 nM for 24 h) displayed higher levels of CRT on their cell surface than untreated HMLER-shEcad cells (Fig. 5A). A similar result was observed for HMLER-shEcad cells treated with 1 (0.2 μM for 24 h) and co-dosed with cisplatin (150 μM for 24 h) and thapsigargin (7 μM for 24 h; positive control) (Fig. S25 and S26†). ATP released from dying cells act as a “find me” signal for immune cells.^15^ ATP secretion from HMLER-shEcad cells treated with 1 NP^10^ (100–200 nM for 24 h), 1 (0.4–0.8 μM for 24 h), and cisplatin (10–20 μM for 24 h, positive control) was determined by analysing the supernatant using a luciferase-based assay (Fig. 5B). A 3.5–3.6-fold increase in extracellular ATP was observed upon 1 NP^10^ treatment. Treatment with the payload 1 and cisplatin also induced significant (*p* < 0.05) ATP release (Fig. 5B). Nuclear HMGB-1 excreted from dying cells upon plasma membrane permeabilisation acts as a cytokine, and promotes antigen processing and presentation to T-cells.^46^ The relative amount of HMGB-1 in HMLER-shEcad cells treated with 1 NP^10^ was assessed by immunoblotting studies to gauge potential HMGB-1 release. HMLER-shEcad cells treated with 1 NP^10^ (95–764 nM for 24 h) displayed markedly lower or undetectable amounts of HMGB-1 relative to untreated control cells, indicative of HMGB-1 excretion (Fig. S27†). A similar result was previously reported for HMLER-shEcad cells treated with 1.^20^ Taken together, the DAMP detection studies show that 1 NP^10^ induces CRT cell surface exposure, ATP release, and intracellular HMGB-1 depletion in breast CSCs, and thus implies that 1 NP^10^-mediated breast CSC death is consistent with ICD (alike the payload 1).^20^

## Conclusion

In summary we report the encapsulation of an ICD-inducing copper(ii) complex 1 into polymeric PEG–PLGA nanoparticles and the anti-CSC properties of the optimal nanoparticle formulation. The most efficient loading conditions were attained when the feed of 1 was fixed at 10%, yielding 1 NP^10^. These conditions produced spherical nanoparticles, which were characterised by DLS and SEM imaging to possess a diameter of *ca.* 108–118 nm. The nanoparticles size is ideally suited to exploit the EPR effect and target tumour microenvironments *in vivo*, which augers well for further translation. The nanoparticle formulation 1 NP^10^ was stable in various biologically relevant solutions over the course of 72 h at 37 °C. This confirmed that the intact cellular delivery of 1 by 1 NP^10^ is feasible. The nanoparticle formulation 1 NP^10^ was taken up by breast CSCs (HMLER-shEcad cells) *via* a temperature-dependent endocytic pathway in significantly greater quantities than the free payload 1. The release profile of 1 NP^10^ indicated that it is able to selectivity release 1 under acidic conditions resembling endosomal compartments. Therefore, 1 NP^10^ is likely to enter CSCs *via* endocytosis and release the majority of its payload only once inside acidic endosomal vesicles.

Notably, 1 NP^10^ was 16-fold and 5.4-fold more potent than 1 toward breast CSCs grown in monolayer and three-dimensional cultures, respectively. The greater breast CSC toxicity of 1 NP^10^ relative to 1 is attributed to the higher internalisation of the nanoparticle formulation compared to the free payload. Strikingly, 1 NP^10^ was 185-fold more toxic towards mammospheres than salinomycin, the most clinically advanced anti-breast CSC agent to date. The nanoparticle formulation 1 NP^10^ was able kill bulk breast cancer cells and breast CSCs within a small concentration window (the IC_50_ value for HMLER and HMLER-shEcad cells in monolayer cultures was within 0.01 μM). Therefore, the nanoparticle formulation 1 NP^10^ retained the potential of the payload to eradicate entire breast cancer cell populations (comprising of bulk cancer cells and CSCs) with a single (nanomolar) dose. Studies aimed at deciphering the mechanism of action showed that 1 NP^10^ is able to elevate intracellular ROS levels, induce ER stress, and prompt all the hallmarks of ICD in breast CSCs. Overall, the mechanistic profile of 1 NP^10^ is similar to that of the payload 1. This is a highly favorable characteristic as the progression of many nanoparticle formulations within the clinical setting is often discontinued based on disparities in the mechanism of action of the payload prior to encapsulation and after encapsulation into nanoparticles. Our results clearly show that polymeric nanoparticles can be used to effectively transport ICD-inducing metal complexes into CSCs (without changing their mechanism of action), and moreover paves the way for the development of other nanoparticle constructs that can impact CSCs in a cytotoxic and immunologically manner.

## Experimental

### Encapsulation of 1 into PEG–PLGA nanoparticles

The nanoprecipitation method was used to encapsulate the ICD-inducing copper(ii) agent 1 into PEG–PLGA nanoparticles. First, 10 mg of PEG–PLGA (5000 : 20 000 Da, 1 : 1 LA : GA) and various amounts of 1 (0.5–5 mg), were dissolved in 0.5 mL of DMF. The amount of 1 used varied accordingly to the desired feed, defined as mg of 1/mg of polymer × 100. The DMF solution was added in a dropwise manner to 5 mL of stirring MilliQ water (0.5 cm magnetic stirrer, 800 rpm rotation speed). The encapsulation reaction was carried out in a 20 mL glass scintillation vial at room temperature. After the addition of the DMF solution (containing the PEG–PLGA polymer and 1) to MilliQ water, the water acquired a milky blue colour due to the Tyndall effect of the nanoparticles formed. At this stage, 4.5 mL of MilliQ water were added to the resultant solution in order to bring the total volume up to 10 mL, and the solution was allowed to stir for an additional 20 minutes at room temperature. The nanoparticle solution was then loaded onto an Amicon Centrifugal Filtration Device (with a regenerated cellulose membrane and a 100 kDa MW cut-off) and centrifuged for 12 minutes at 2000 rpm speed (at 18 °C). The concentrated solution was diluted with 10 mL of MilliQ water and centrifuged further under the aforementioned conditions. This was repeated three times to ensure any unencapsulated 1 was removed. The final concentrated suspension was diluted to 1 mL with MilliQ water and filtered to remove any aggregates (a filter with a cut-off of 0.2 μm was used). The filtered suspension was diluted further with MilliQ water and used for further experiments. The amount of copper present in the final suspension was measured by ICP-MS (ThermoScientific ICAP-Qc quadrupole). The measured copper content was used to calculate the loading efficiency and encapsulation efficiency; the amount of copper present in the final nanoparticle formulation relative to the amount of polymer (loading efficiency) or 1 (encapsulation efficiency) used × 100. Empty PEG–PLGA nanoparticles were prepared using the above method without the addition of 1, and used as a control. In this case, it was assumed that all of the PEG–PLGA polymer used (10 mg) formed nanoparticles.

### Dynamic light scattering and scanning electron microscope

The nanoparticle size distribution and polydispersity was obtained by loading aqueous solutions of the nanoparticle formulation 1 NP^10^ into disposable micro-cuvettes and measuring the dynamic light scattering (DLS) of the solution using a Zetasizer Nanoseries spectrometer (Malvern). For the Scanning Electron Microscope (SEM) studies, an aliquot of 1 NP^10^ in MilliQ water was allowed to evaporate on a square glass slide and coated with platinum. Imaging was conducted using a Hitachi S4000 Scanning Electron Microscope within the University of Leicester Advanced Microscopy Facility.

### Payload release studies

The nanoparticle formulation 1 NP^10^ was incubated in sodium acetate buffer (pH 5.2) or PBS (pH 7.4) for 72 h at 37 °C. At specific time points over the course of the incubation period, the nanoparticle solution was removed and passed through an Amicon Centrifugal Filter (with a 100 kDa MW cut-off) and replenished with fresh sodium acetate buffer (pH 5.2) or PBS (pH 7.4). The copper content of the filtrates obtained at each of the time points was measured by ICP-MS and used to calculate the percentage of payload released.

### General cell culture conditions

HMLER and HMLER-shEcad cells derived from normal mammary epithelial cells, were gifted to us by Prof. R. A. Weinberg (Whitehead Institute, MIT). The cells were cultured using Mammary Epithelial Cell Growth Medium (MEGM) containing BPE, hydrocortisone, hEGF, insulin, and gentamicin/amphotericin-B (Lonza). The cells were handled in a sterile environment at all times and cultured in an incubator that was maintained at 37 °C, with an internal atmosphere containing 5% CO_2_.

### Cellular uptake

Cellular uptake studies involving the nanoparticle formulation 1 NP^10^ and payload 1 were conducted under various conditions. HMLER and HMLER-shEcad cells (*ca.* 1 million) were treated with 1 NP^10^ and 1 (110 nM) at 4 °C or 37 °C for 4 h or 24 h. In the case of 1 NP^10^, experiments were also conducted in the presence of endocytosis inhibitors, NH_4_Cl (50 mM) and chloroquine (100 μM). After incubation, the media containing the nanoparticle formulation 1 NP^10^ or payload 1 (with or without the endocytosis inhibitors) was aspirated and the remaining adherent cells were thoroughly washed with 2 mL of PBS, three times. The cells were then collected by trypsinisation and centrifuged to form a pellet. The resultant pellet was digested with 65% HNO_3_ (250 μL) overnight at room temperature. Cellular pellets obtained from HMLER-shEcad cells treated with 1 NP^10^ (110 nM at 37 °C for 24 h) were also used to determine the intracellular distribution of 1 NP^10^. For this, the nuclear, cytoplasmic, and membrane fractions were isolated using the Thermo Scientific NE-PER Nuclear and Cytoplasmic Extraction Kit. The extracted nuclear, cytoplasmic, and membrane fractions were digested with 65% HNO_3_ (250 μL) overnight at room temperature. All of the cellular material digested by 65% HNO_3_ were diluted with MilliQ water and measured by ICP-MS to determine the copper content (Thermo Scientific iCAP-Qc quadrupole). The copper content in each sample (cellular material) is represented as Cu (ng) per million cells (overall *n* = 4).

### Cytotoxicity MTT assay

The colorimetric MTT assay was used to determine the toxicity of the empty PEG–PLGA nanoparticle and 1 NP^10^. HMLER and HMLER-shEcad cells (5 × 10^3^) were seeded in each well of a 96-well plate. After incubating the cells overnight, various concentrations of the empty PEG–PLGA nanoparticle and 1 NP^10^, as determined by ICP-MS, were added and incubated for 72 h (total volume 200 μL). After the incubation period, a PBS solution containing MTT (4 mg mL^−1^) was added to each well of the 96-well plate. Specifically, 20 μL of the PBS-MTT solution was added. After the addition, the 96-well plates were incubated for 4 h. The solution was then removed from each well to leave behind purple formazan crystals. The purple formazan crystals were dissolved in DMSO (200 μL), and the absorbance of the solution was measured using a plate reader at 550 nm. The absorbance of the solutions in each well were normalised to untreated control wells, and used to generate dose–response curves with concentration of test agent on the *x*-axis and % HMLER or HMLER-shEcad cell viability on the *y*-axis. The IC_50_ values, the concentration required to reduce cell viability by half, were interpolated from the dose dependent curves. The cytotoxicity MTT assay was repeated three time per test agent, per cell line. In each experiment, each concentration tested was repeated six times (overall *n* = 18).

### Tumorsphere formation and viability assay

HMLER-shEcad (5 × 10^3^ cells per well) were plated in ultralow-attachment 96-well plates (Corning) and incubated in MEGM supplemented with B27 (Invitrogen), 20 ng mL^−1^ EGF, and 4 μg mL^−1^ heparin (Sigma) for 5 days. Studies were also conducted in the presence of the empty PEG–PLGA nanoparticle, 1 NP^10^, 1, or salinomycin. Mammospheres treated with the empty PEG–PLGA nanoparticle, 1 NP^10^, 1, and salinomycin (at their respective IC_20_ values for 5 days) were imaged and counted using a standard inverted microscope. The TOX8 solution (20 μL, Sigma) was added to each well to determine the viability of the mammospheres. After the addition, the 96-well plates were incubated for 16 h. The fluorescence of the solution was measured using a plate reader at 590 nm (*λ*_ex_ = 560 nm). The fluorescence of the solutions in each well were normalised to untreated control wells, and used to generate dose–response curves with concentration of test agent on the *x*-axis and % HMLER-shEcad mammosphere viability on the *y*-axis. The IC_50_ values, the concentration required to reduce HMLER-shEcad mammosphere viability by 50%, were interpolated from the dose–response curves. The HMLER-shEcad mammosphere viability assay using TOX8 was repeated three time per test agent. In each experiment, each concentration tested was repeated two times (overall *n* = 6).

### Intracellular ROS detection assay

HMLER-shEcad cells (5 × 10^3^ cells) were plated into every well of a 96-well plate. The cells were left to attach overnight, after which the cells were dosed with the nanoparticle formulation 1 NP^10^ (IC_50_ value) for 24 h. At specific time points over the course of the 24 h incubation period, the intracellular ROS levels were measured using 2′,7′-dichlorodihydrofluorescein diacetate. Specifically, 2′,7′-dichlorodihydrofluorescein diacetate (20 μM) was added to each well (to be measured) and incubated for 30 min, then the ROS was detected using a plate reader by measuring the fluorescence of the solutions in the wells at 529 nm (*λ*_ex_ = 504 nm).

### Western blot analysis

HMLER-shEcad cells (5 × 10^5^ cells) were plated in 60 mm Petri dishes and incubated overnight. The cells were then dosed with the nanoparticle formulation 1 NP^10^ (37–191 nM for 4 h, 24 h or 72 h). After the treatment period, the media was removed and the cells were washed with PBS. The cells were then collected using the SDS-PAGE loading buffer (64 mM Tris–HCl (pH 6.8)/9.6% glycerol/2%SDS/5% β-mercaptoethanol/0.01% bromophenol blue) and the cell suspension was heated to 95 °C for 10 min to fully denature the content. The cell lysates were then loaded onto 4–20% sodium dodecylsulphate polyacylamide gels and subject to electrophoresis (SDS-PAGE; 200 V for 25 min). The separated proteins were transferred onto a polyvinylidene difluoride membranes, PVDF (350 mA for 1 h) using standard immunoblotting methods. The PVDF membranes were blocked with 5% non-fat dry milk for 30 min prior to incubation with various primary antibodies (Cell Signalling Technology) overnight at 4 °C. The membranes were then washed once with PBS and three times with PSBT (1% Tween 20). The PVDF membranes were then incubated with horseradish peroxidase-conjugated secondary antibodies (Cell Signalling Technology) for 1 h at room temperature. The membranes were again washed once with PBS and three times with PSBT (1% Tween 20). Then the antibody complexes on the PVDF membranes were detected with the ECL detection reagent (hydrogen peroxide and luminol, BioRad) and imaged using an imager with chemiluminescence capabilities (Bio-Rad ChemiDoc Imaging System).

### CRT cell surface exposure

Flow cytometry was used to analyse cell surface CRT exposure. HMLER-shEcad cells were seeded into a 6-well plate (at a density of 5 × 10^5^ cells per mL) and the cells were incubated at 37 °C overnight. The cells were treated with 1 NP^10^ (46–371 nM) or 1 (0.2 μM) or co-treated with cisplatin (150 μM) with thapsigargin (7 μM) for 24 hours at 37 °C. The cells were then harvested by trypsinisation and collected by centrifugation. The pellet was suspended in PBS (500 μL), and after the addition of the Alexa Fluor® 488 nm labelled anti-CRT antibody (5 μL), the cells were incubated in the dark for 30 minutes. The cells were then washed with PBS (1 mL) and analysed using a FACSCanto II flow cytometer (BD Biosciences) (10 000 events per sample were acquired). The FL1 channel was used to assess CRT cell surface exposure. Cell populations were analysed using the FlowJo software (Tree Star).

### ATP assay

HMLER-shEcad cells (5 × 10^3^ cells per well) were seeded in a 96-well plate and incubated overnight. The cells were then treated with 1 NP^10^ (100–200 nM for 24 h), 1 (0.4–0.8 μM for 24 h), or cisplatin (10–20 μM for 24 h) at 37 °C. The media was carefully extracted and transferred into a white-walled opaque 96-well plate, and the luciferin-based ENLITEN ATP Assay Kit (Promega) was used to measure the relative amount of ATP released into the supernatant.

### HMGB-1 release

HMLER-shEcad cells (5 × 10^5^ cells) were incubated with 1 NP^10^ (95–764 nM for 24 h) at 37 °C to induce cell death. Cells were collected in full and added to SDS-PAGE loading buffer and incubated at 95 °C for 10 min. Intracellular HMGB-1 was detected by western blot analysis using the cell lysates and the anti-HMGB-1 antibody (Cell Signalling Technology).

## Author contributions

Conceptualization, K. S. and G. P.; methodology, K. S., G. P. and J. N. S.; software, K. S., G. P. and J. N. S.; validation, K. S., G. P. and J. N. S.; formal analysis, K. S., G. P. and J. N. S.; investigation, K. S., G. P. and J. N. S.; resources, K. S.; data curation, K. S., G. P. and J. N. S.; writing—original draft preparation, K. S. and G. P.; writing—review and editing, K. S., G. P. and J. N. S.; visualization, K. S., G. P. and J. N. S.; supervision, K. S.; project administration, K. S.; funding acquisition, K. S.

## Conflicts of interest

There are no conflicts to declare.

## Supplementary Material

RA-012-D1RA08788F-s001

RA-012-D1RA08788F-s002

RA-012-D1RA08788F-s003

RA-012-D1RA08788F-s004

RA-012-D1RA08788F-s005

RA-012-D1RA08788F-s006

RA-012-D1RA08788F-s007

RA-012-D1RA08788F-s008

RA-012-D1RA08788F-s009

RA-012-D1RA08788F-s010

RA-012-D1RA08788F-s011

RA-012-D1RA08788F-s012

RA-012-D1RA08788F-s013
