# Supplementary figures and images for: Delivery of an immunogenic cell death-inducing copper complex to cancer stem cells using polymeric nanoparticles
Source: RSC Adv. 2022 Feb 11;12(9):5290–9. doi: 10.1039/d1ra08788f (PMC8981415; doi:10.1039/d1ra08788f)

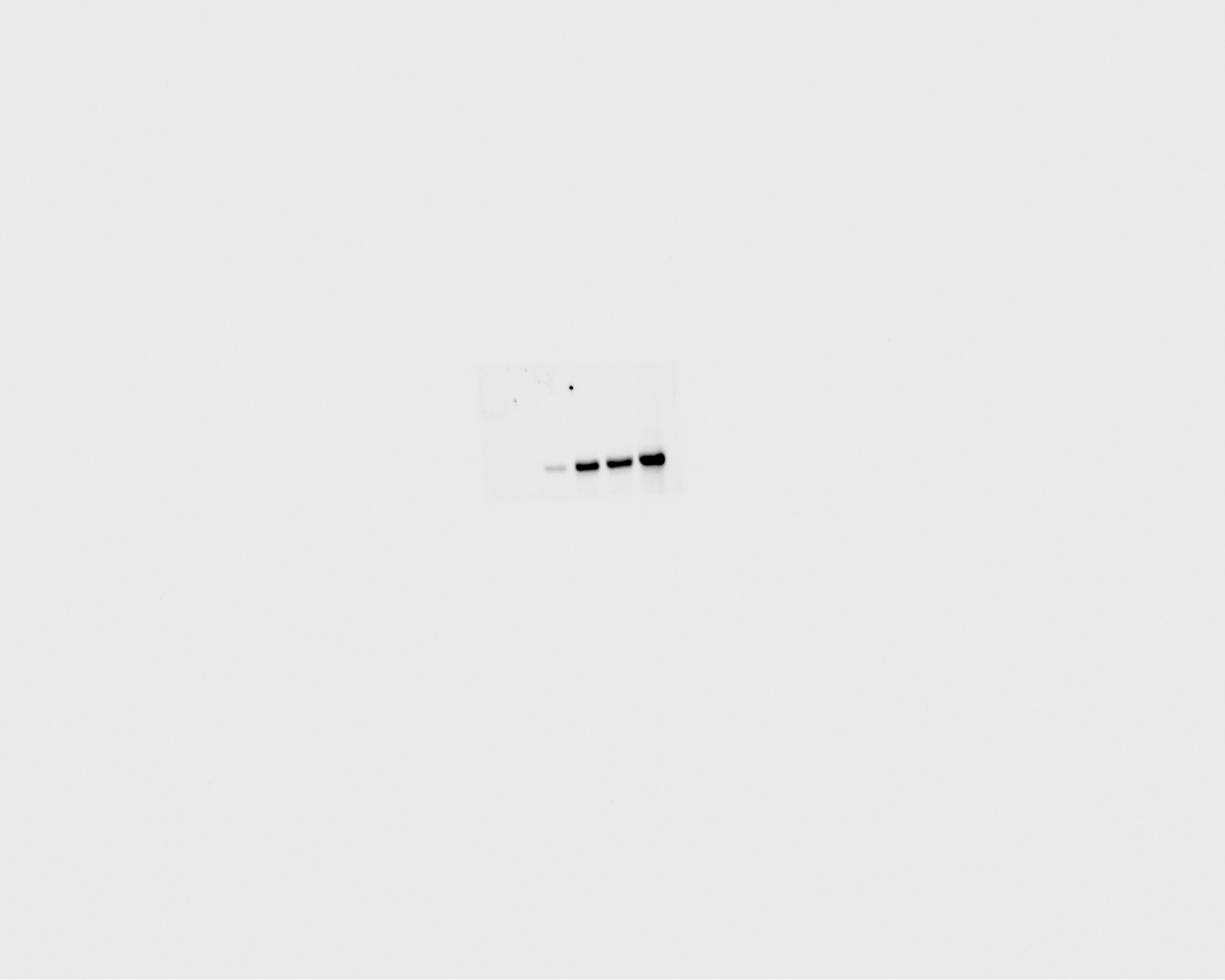

Supplement: RA-012-D1RA08788F-s001 [file RA-012-D1RA08788F-s001.tif]

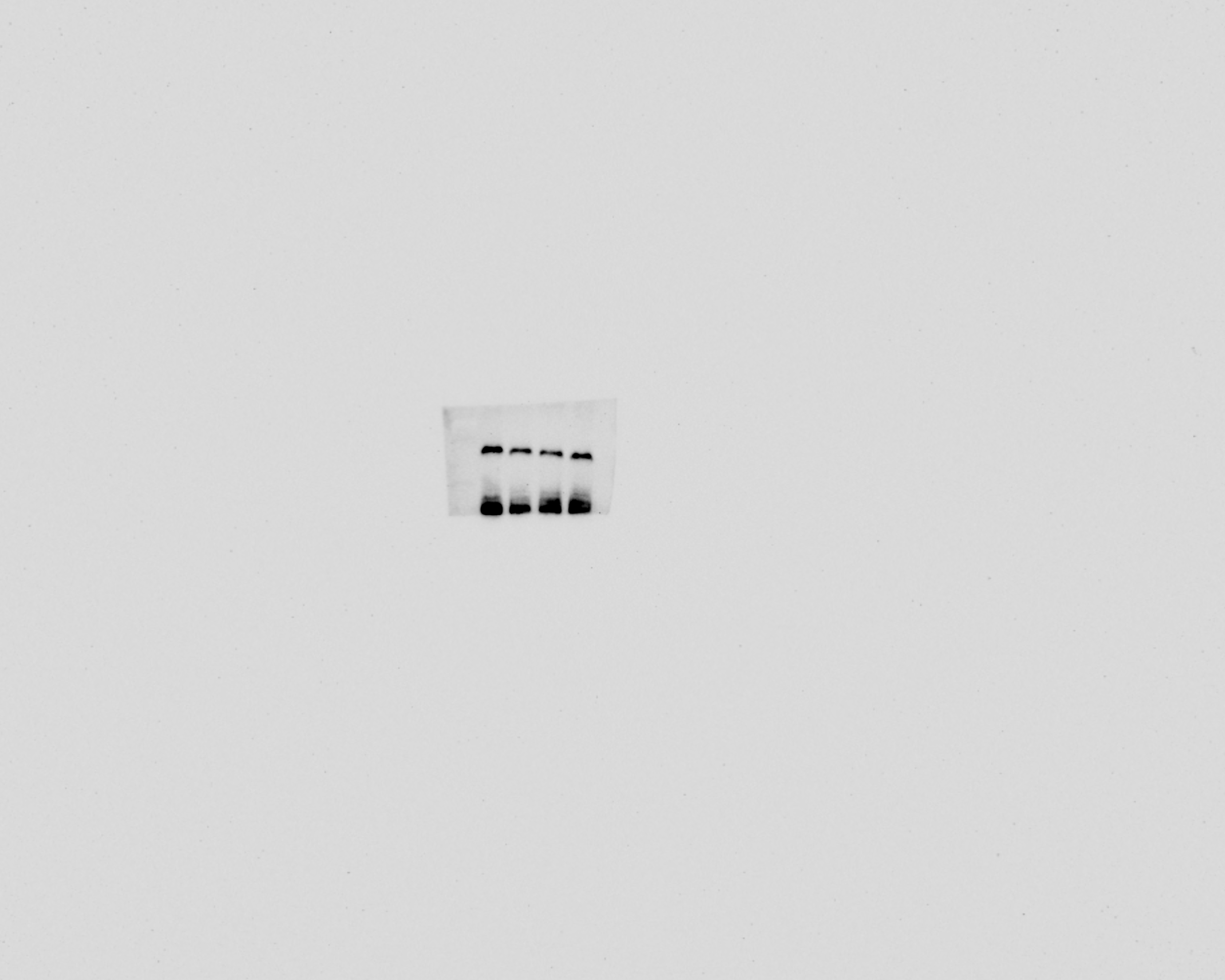

Supplement: RA-012-D1RA08788F-s002 [file RA-012-D1RA08788F-s002.tif]

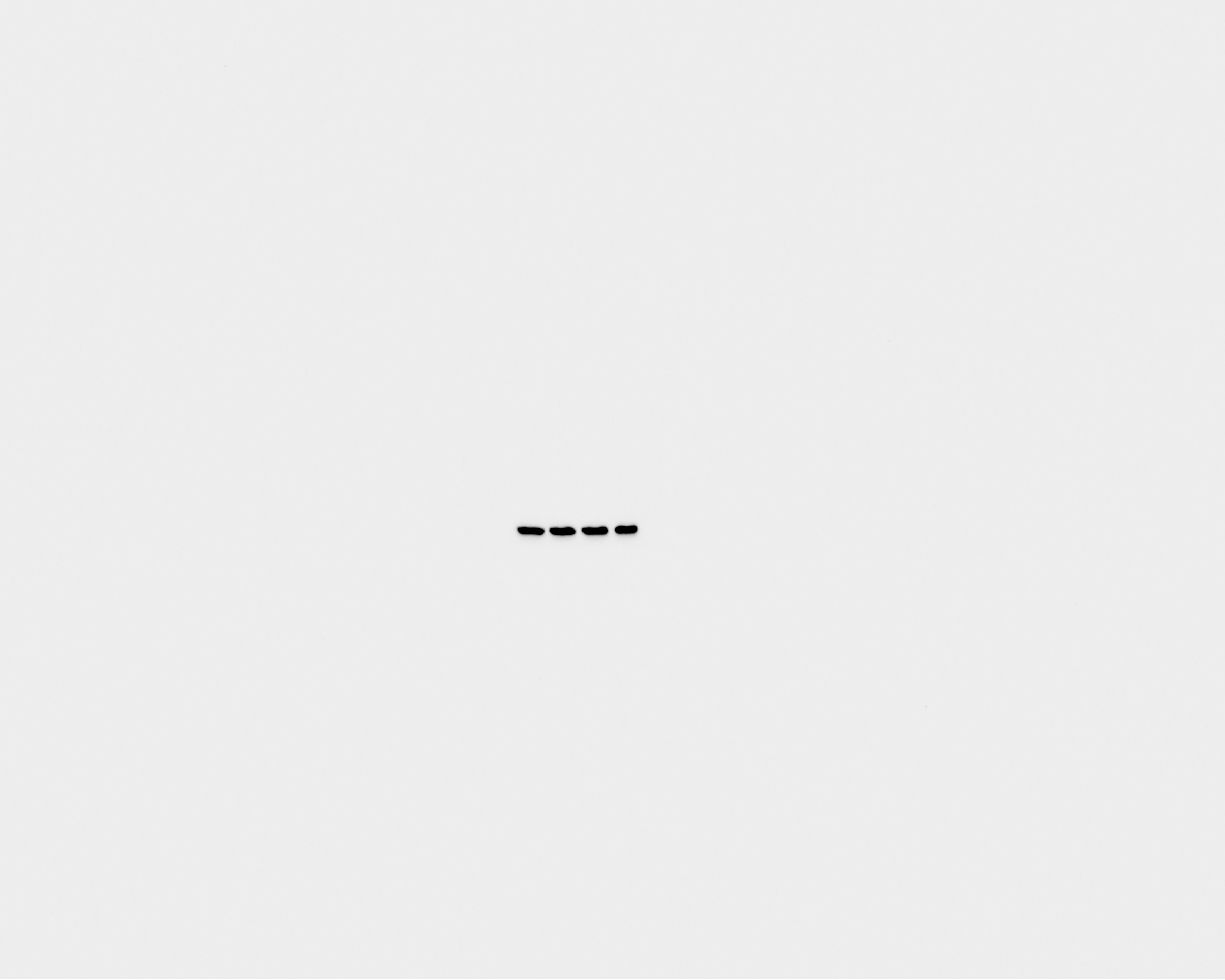

Supplement: RA-012-D1RA08788F-s003 [file RA-012-D1RA08788F-s003.tif]

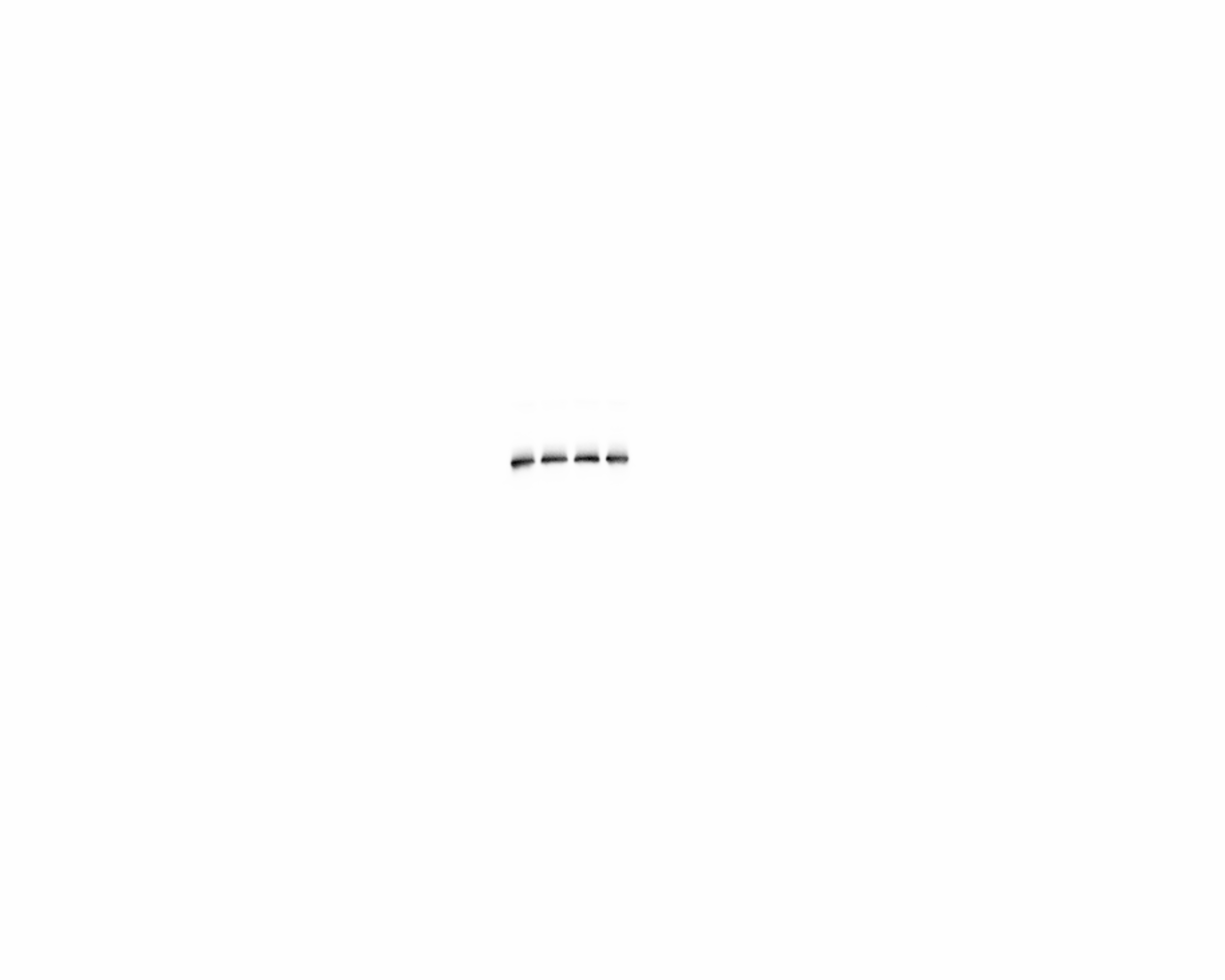

Supplement: RA-012-D1RA08788F-s004 [file RA-012-D1RA08788F-s004.tif]

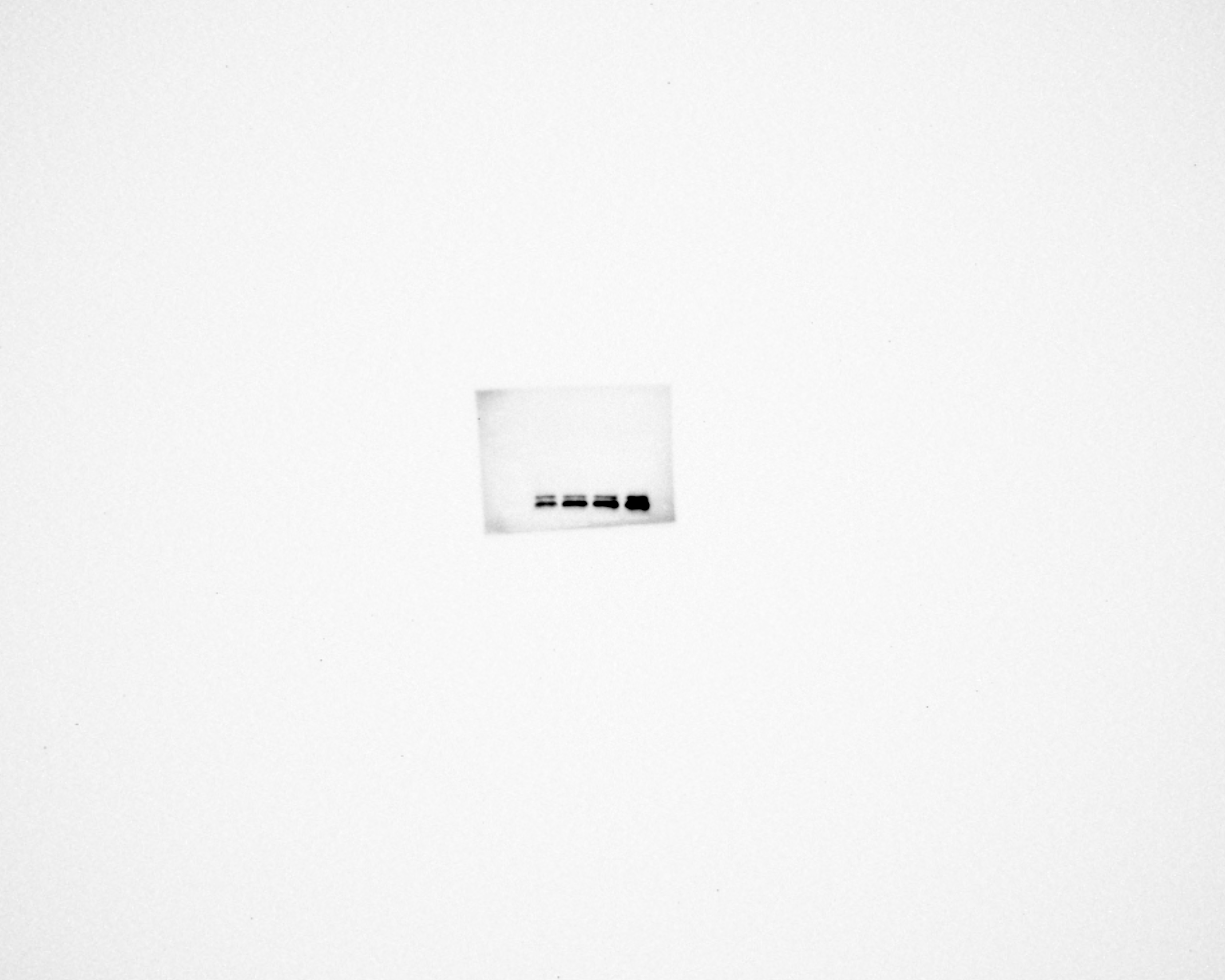

Supplement: RA-012-D1RA08788F-s005 [file RA-012-D1RA08788F-s005.tif]

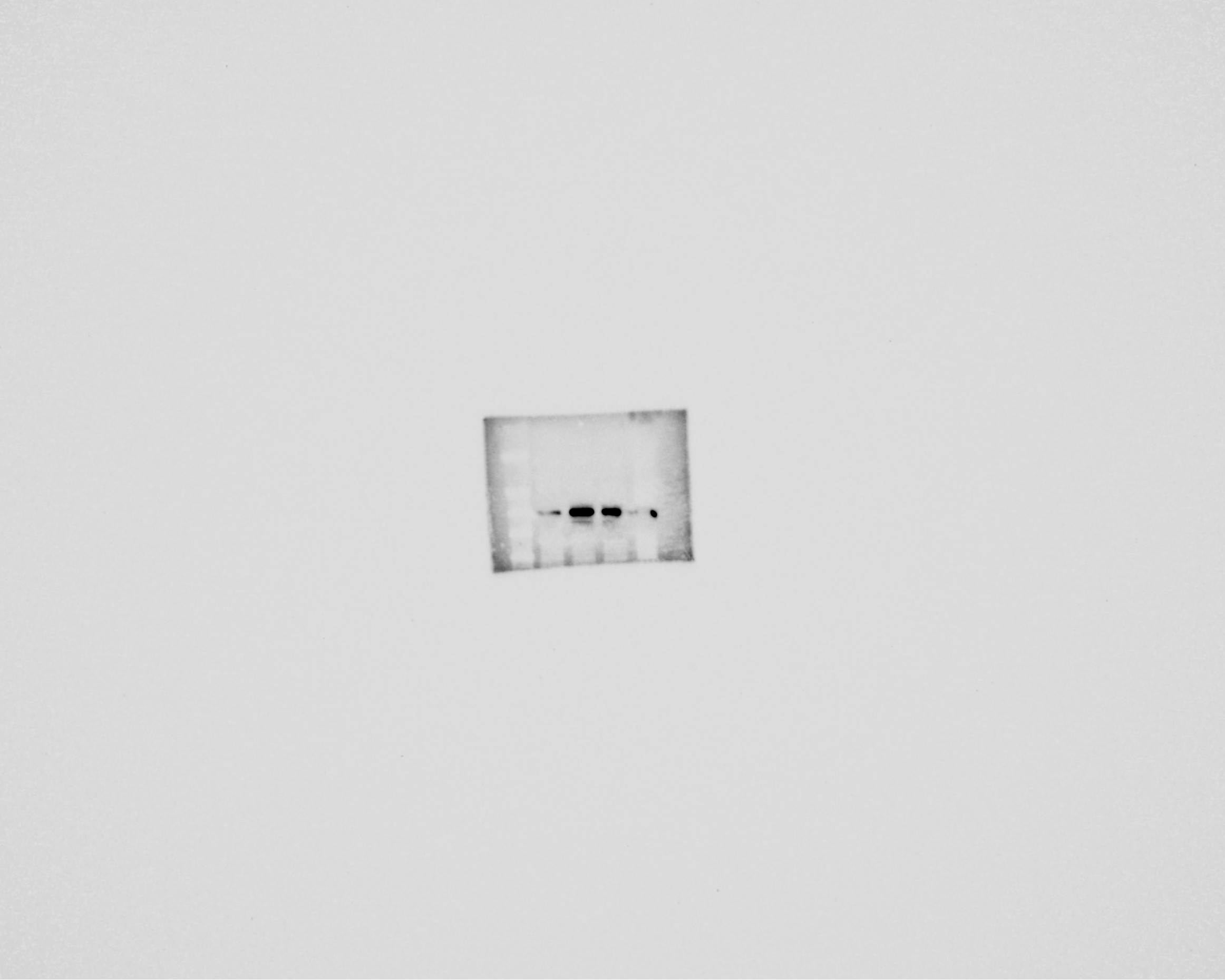

Supplement: RA-012-D1RA08788F-s006 [file RA-012-D1RA08788F-s006.tif]

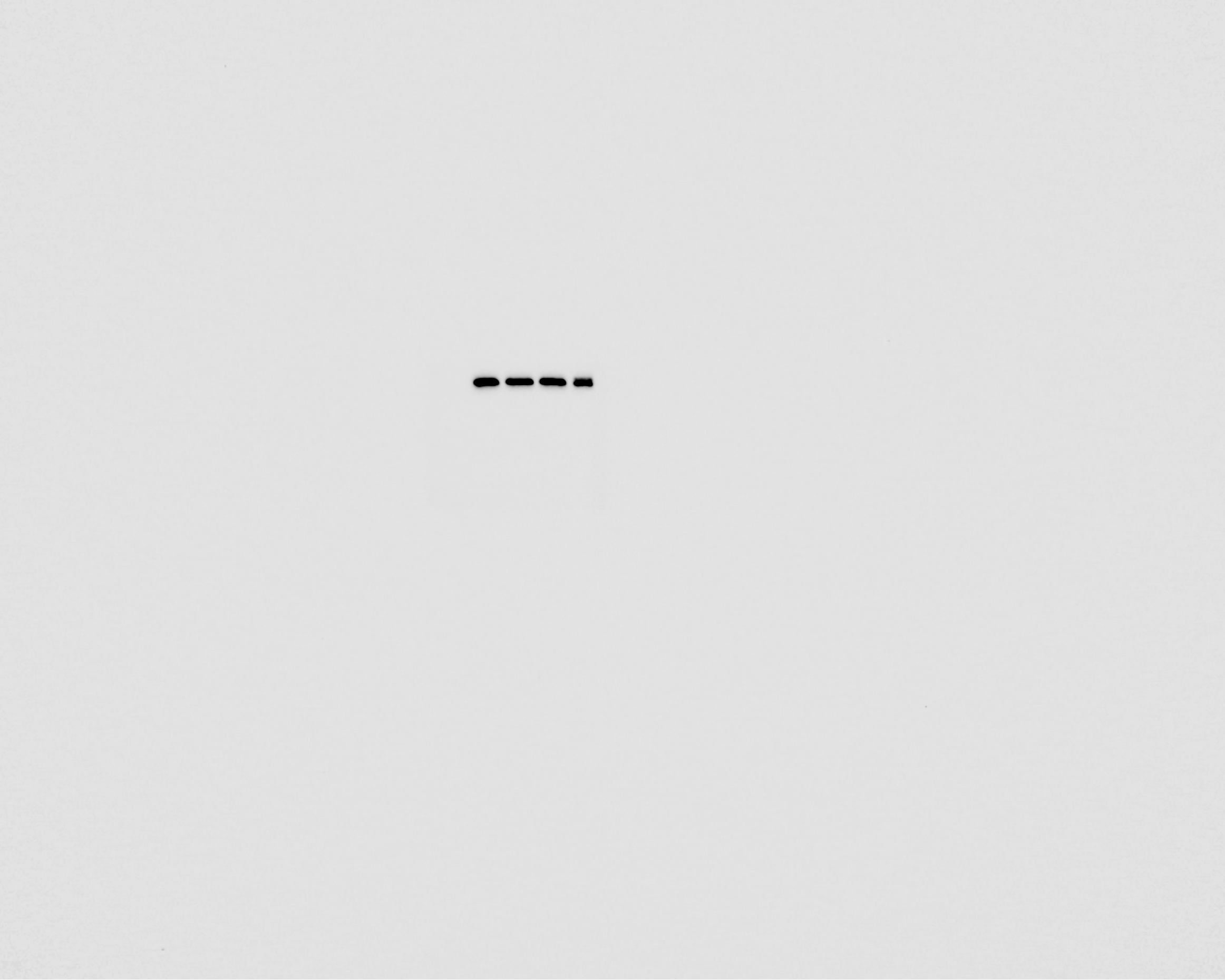

Supplement: RA-012-D1RA08788F-s007 [file RA-012-D1RA08788F-s007.tif]

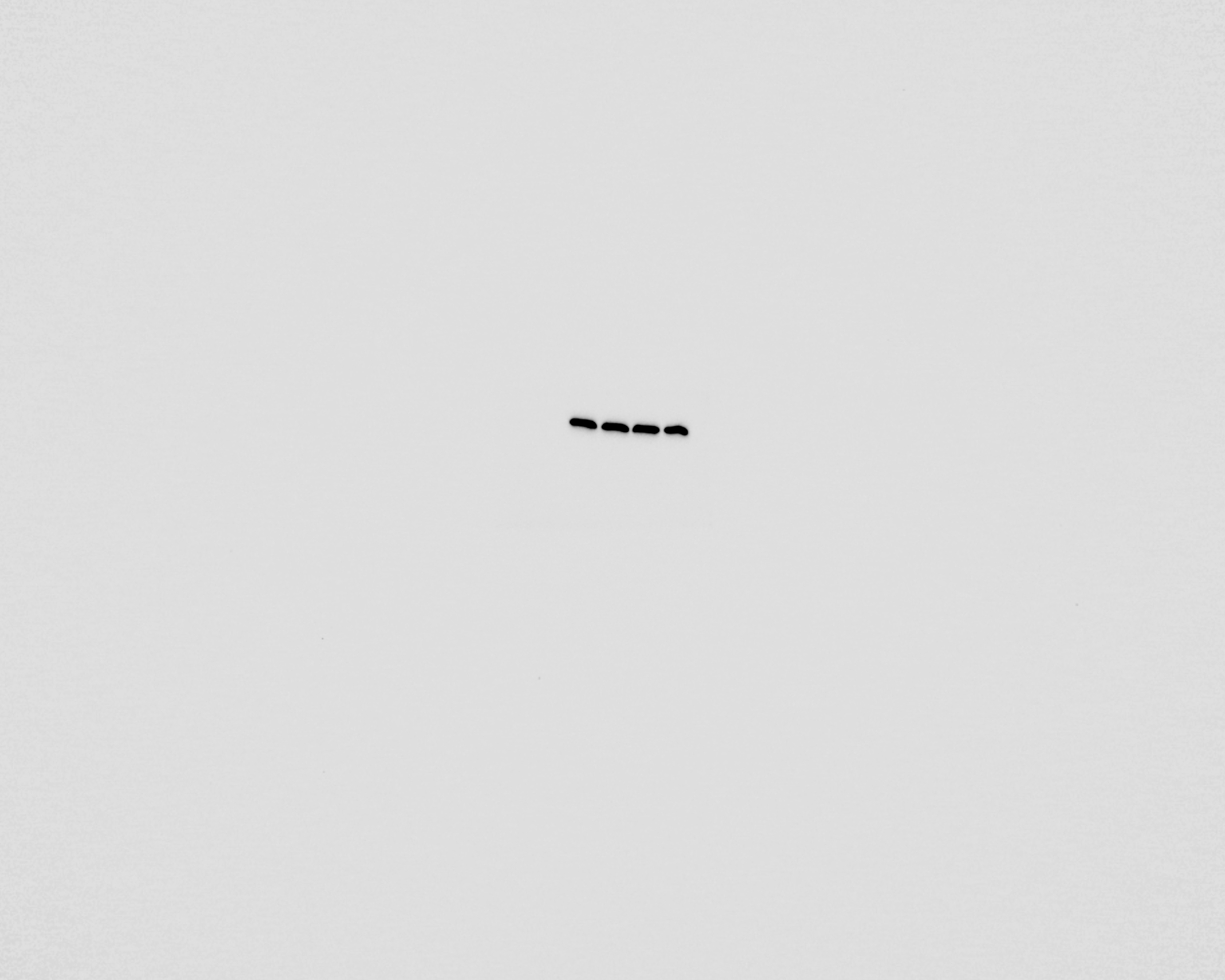

Supplement: RA-012-D1RA08788F-s008 [file RA-012-D1RA08788F-s008.tif]

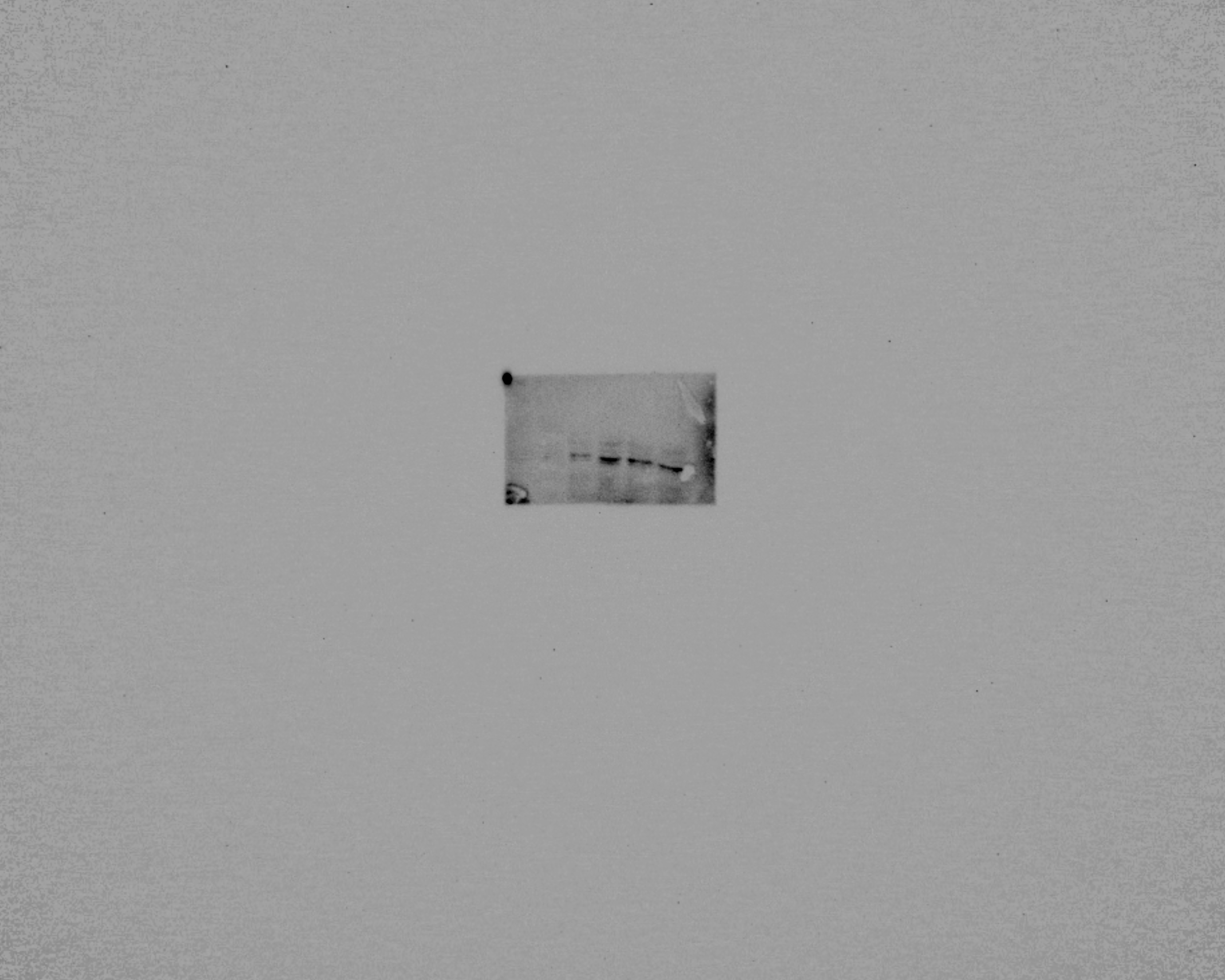

Supplement: RA-012-D1RA08788F-s009 [file RA-012-D1RA08788F-s009.tif]

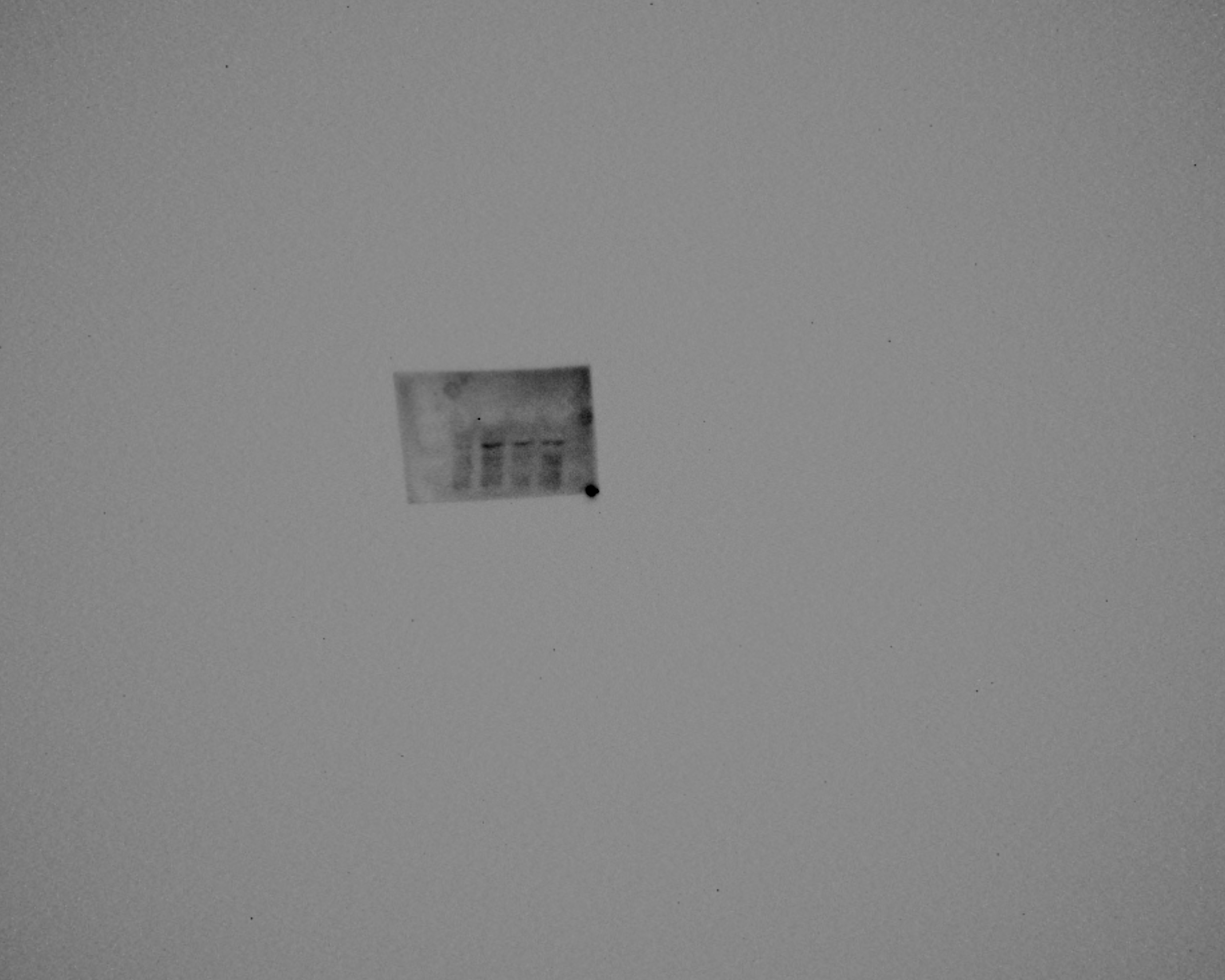

Supplement: RA-012-D1RA08788F-s010 [file RA-012-D1RA08788F-s010.tif]

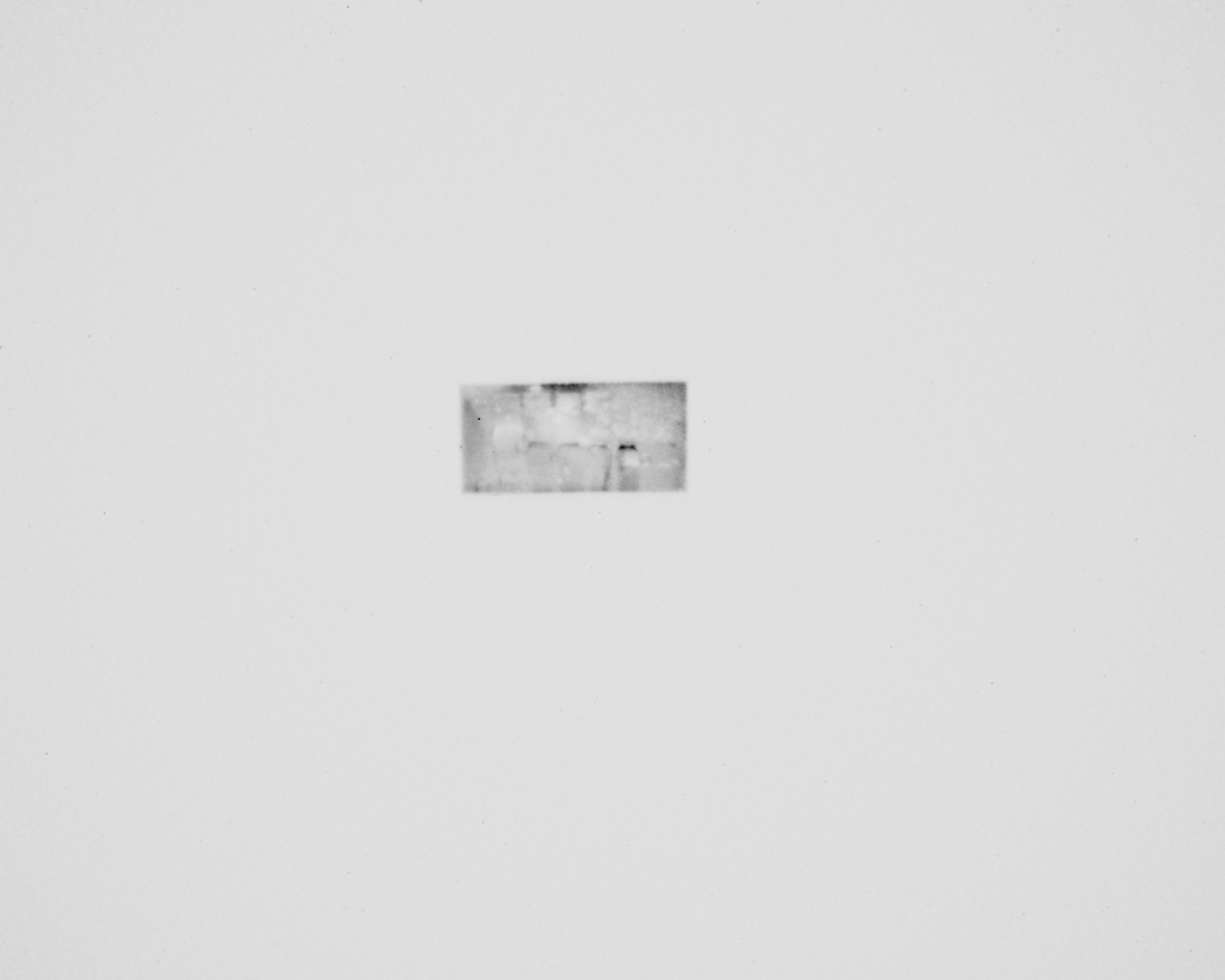

Supplement: RA-012-D1RA08788F-s011 [file RA-012-D1RA08788F-s011.tif]

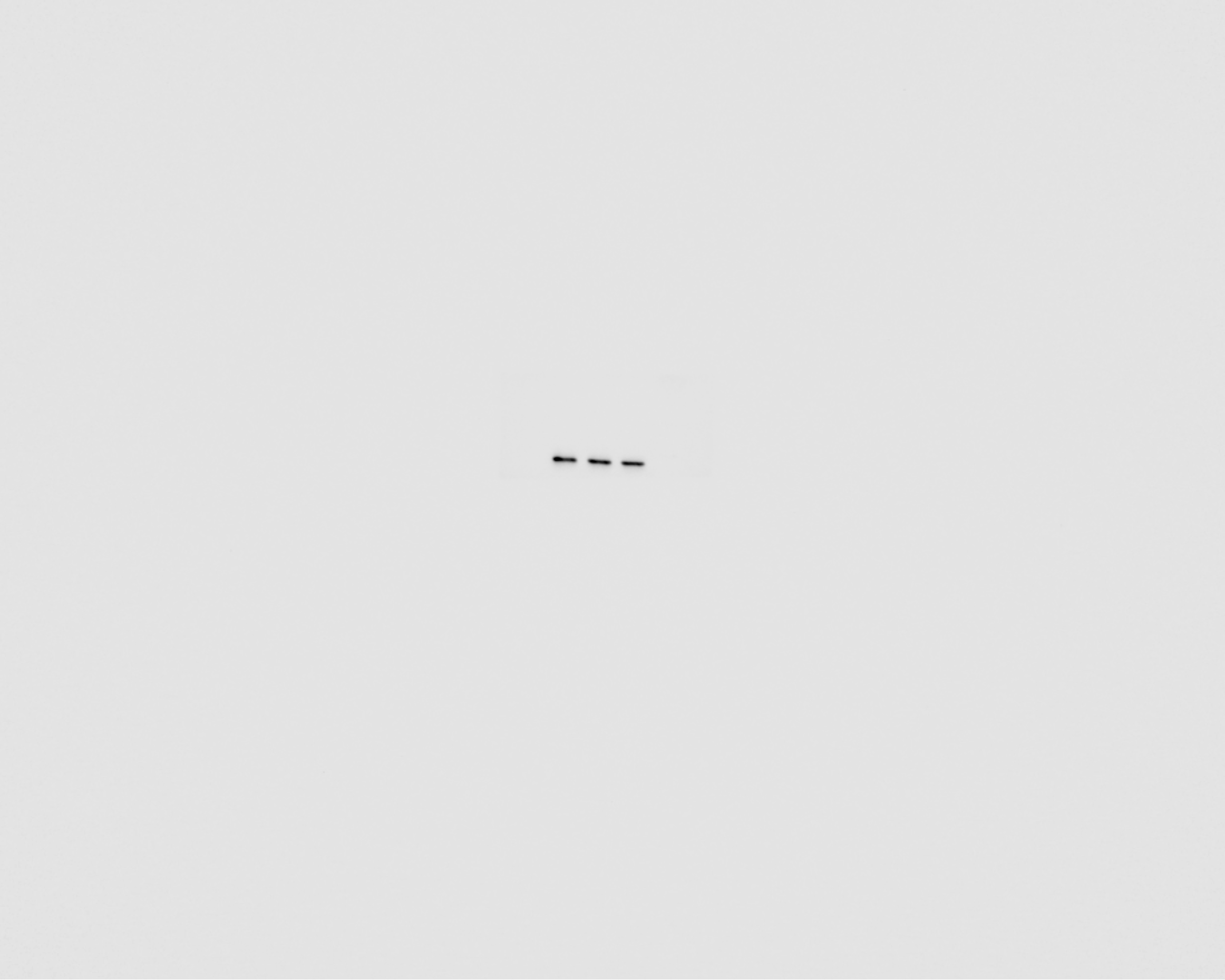

Supplement: RA-012-D1RA08788F-s012 [file RA-012-D1RA08788F-s012.tif]
