# Supplementary material for: Delivery of an immunogenic cell death-inducing copper complex to cancer stem cells using polymeric nanoparticles
Source: RSC Adv. 2022 Feb 11;12(9):5290–9. doi: 10.1039/d1ra08788f (PMC8981415; doi:10.1039/d1ra08788f)
Supplement: RA-012-D1RA08788F-s013 [file RA-012-D1RA08788F-s013.pdf]

Electronic Supplementary Material (ESI) for RSC Advances.
This journal is © The Royal Society of Chemistry 2022

Supporting Information for

Delivery of an Immunogenic Cell Death-Inducing Copper Complex to Cancer Stem Cells Using Polymeric Nanoparticles

Ginevra Passeri [1], Joshua Northcote-Smith [1], and Kogularamanan Suntharalingam [1,*]

[1] School of Chemistry, University of Leicester, Leicester, LE1 7RH, United Kingdom

Email: k.suntharalingam@leicester.ac.uk

Table of Content

**Fig. S1**	UV-Vis spectrum of **1** (25 μM) in DMSO over the course of 24 h at 37 °C.
**Fig. S2**	UV-Vis spectrum of **1** (25 μM) in DMF over the course of 24 h at 37 °C.
**Fig. S3**	UV-Vis spectrum of **1** (25 μM) in PBS:DMSO (200:1) with 10% FBS over the course of 24 h at 37 °C.
**Fig. S4**	UV-Vis spectrum of **1** (25 μM) in PBS:DMSO (200:1) in the presence of ascorbic acid (250 μM) over the course of 24 h at 37 °C.
**Fig. S5**	UV-Vis spectrum of **1** (25 μM) in PBS:DMSO (200:1) in the presence of glutathione (250 μM) over the course of 24 h at 37 °C.
**Fig. S6**	UV-Vis spectrum of **1** (50 μM) in the presence of ascorbic acid (500 μM) and bathocuproine disulfonate, BCS (100 μM) in PBS:DMSO (200:1) over the course of 24 h at 37 °C.
**Fig. S7**	UV-Vis spectrum of **1** (50 μM) in the presence of glutathione (500 μM) and bathocuproine disulfonate, BCS (100 μM) in PBS:DMSO (200:1) over the course of 24 h at 37 °C.
**Fig. S8**	ESI mass spectra (positive mode) of **1** (500 μM) in H_2O:DMSO (10:1) (A), and in the presence of ascorbic acid (5 mM) (B) or in the presence of glutathione (5 mM) (C) after incubation for 24 h at 37 °C.
**Fig. S9**	Dynamic light scattering size distribution of **1 NP[10]** suspended in water. Size refers to diameter of nanoparticles in nm.
**Fig. S10**	Dynamic light scattering size distribution of empty PEG–PLGA nanoparticles suspended in water. Size refers to diameter of nanoparticles in nm.

**Fig. S11**	Variation in **1 NP^{10}** diameter upon incubation in water, PBS with 10% FBS, and mammary epithelial growth medium (MEGM) over the course of 72 h at 37 ^{o}C.
**Fig. S12**	Copper content in HMLER and HMLER-shEcad cells treated with **1 NP^{10}** (110 nM for 24 h) or **1** (110 nM for 24 h) at 37 ^{o}C.
**Fig. S13**	Copper content in HMLER and HMLER-shEcad cells treated with **1 NP^{10}** (110 nM for 4 h) at 4 ^{o}C or 37 ^{o}C.
**Fig. S14**	Copper content in HMLER-shEcad cells treated with **1 NP^{10}** only (16 nM for 24 h), and upon pre-incubation with ammonium chloride (50 mM for 2 h) or chloroquine (100 μM for 2 h) at 37 ^{o}C. Error bars represent standard deviations and Student *t test*, * = $p < 0.05$.
**Fig. S15**	The amount of copper released from **1 NP^{10}** upon incubation in PBS (pH 7.4) or sodium acetate buffer (pH 5.2) over the course of 72 h at 37 ^{o}C.
**Fig. S16**	Representative dose-response curves for the treatment of HMLER and HMLER-shEcad cells with **1 NP^{10}**.
**Fig. S17**	Representative dose-response curves for the treatment of HMLER and HMLER-shEcad cells with empty PEG–PLGA nanoparticles.
**Fig. S18**	Representative bright-field images (× 10) of HMLER-shEcad mammospheres in the absence and presence of salinomycin at its respective IC_{20} values for 5 days.
**Fig. S19**	Representative dose-response curve for the treatment of HMLER-shEcad mammospheres with **1 NP^{10}**.
**Fig. S20**	Representative dose-response curve for the treatment of HMLER-shEcad mammospheres with empty PEG–PLGA nanoparticles.
**Fig. S21**	Representative dose-response curves for the treatment of HMLER-shEcad cells with **1 NP^{10}** after 72 h incubation in the presence of salubrinal (10 μM).
**Fig. S22**	Immunoblotting analysis of proteins related to the unfolded protein response (UPR). Protein expression in HMLER-shEcad cells following treatment with **1 NP^{10}** (40–191 nM) for (A) 4 h or (B) 24 h.
**Fig. S23**	Immunoblotting analysis of proteins related to apoptosis. Protein expression in HMLER-shEcad cells following treatment with **1 NP^{10}** (37–146 nM for 72 h).
**Fig. S24**	Representative dose-response curves for the treatment of HMLER-shEcad cells with **1 NP^{10}** after 72 h incubation in the presence of z-VAD-FMK (5 μM).
**Fig. S25**	Representative histograms displaying the green fluorescence emitted by anti-CRT Alexa Fluor 488 nm antibody-stained HMLER-shEcad cells untreated (red), and treated with **1** (0.2 μM for 24 h) (blue).
**Fig. S26**	Representative histograms displaying the green fluorescence emitted by anti-CRT Alexa Fluor 488 nm antibody-stained HMLER-shEcad cells untreated (red), and treated with cisplatin (150 μM for 24 h) and thapsigargin (7 μM for 24 h) (blue).
**Fig. S27**	Immunoblotting analysis of high mobility group box 1 (HMGB-1). Protein expression in HMLER-shEcad cells following treatment with **1 NP^{10}** (95–764 nM for 24 h).

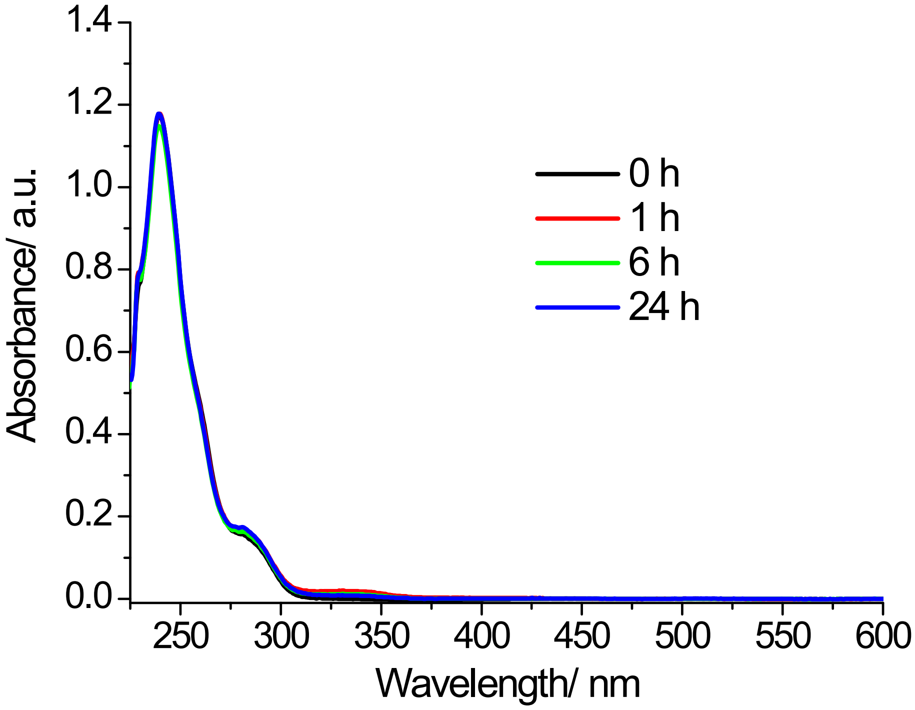

Fig. S1 UV-Vis spectrum of **1** (25 μM) in DMSO over the course of 24 h at 37 °C.

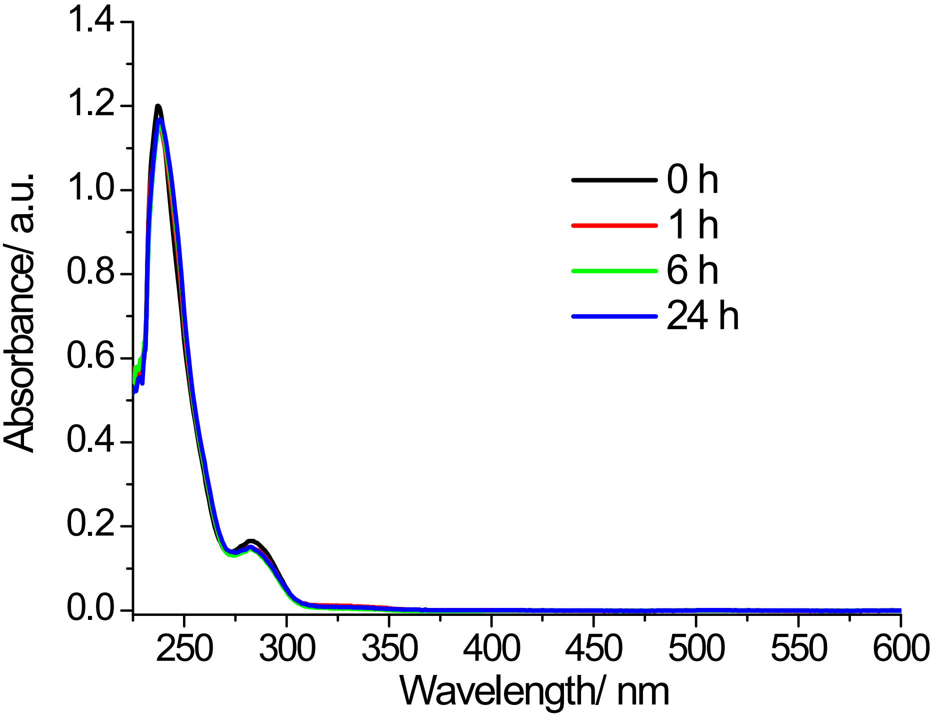

Fig. S2 UV-Vis spectrum of **1** (25 μM) in DMF over the course of 24 h at 37 °C.

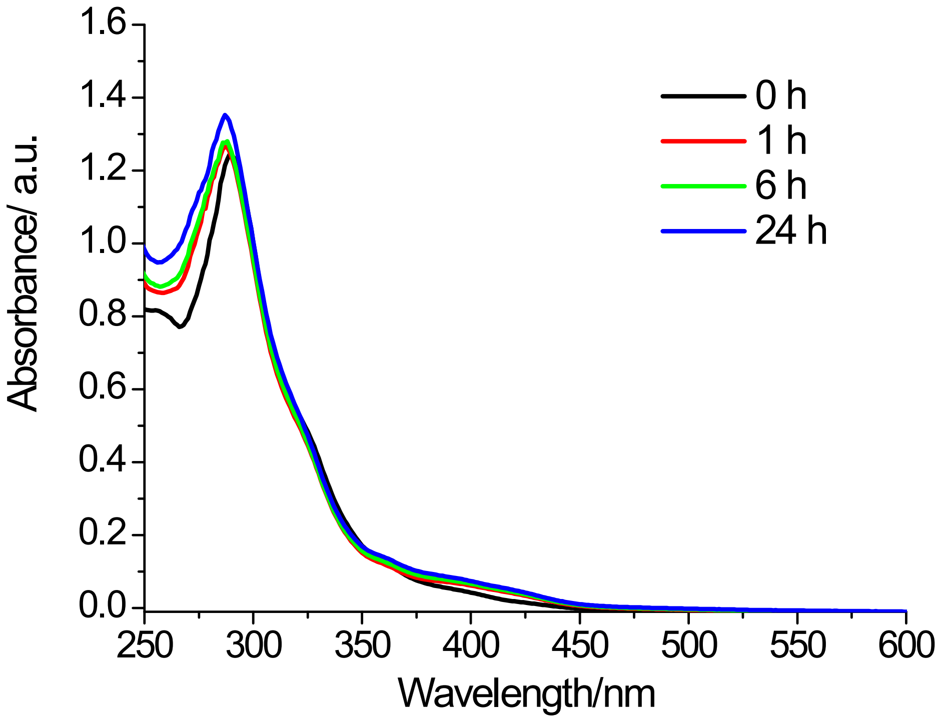

Fig. S3 UV-Vis spectrum of **1** (25 μM) in PBS:DMSO (200:1) with 10% FBS over the course of 24 h at 37 °C.

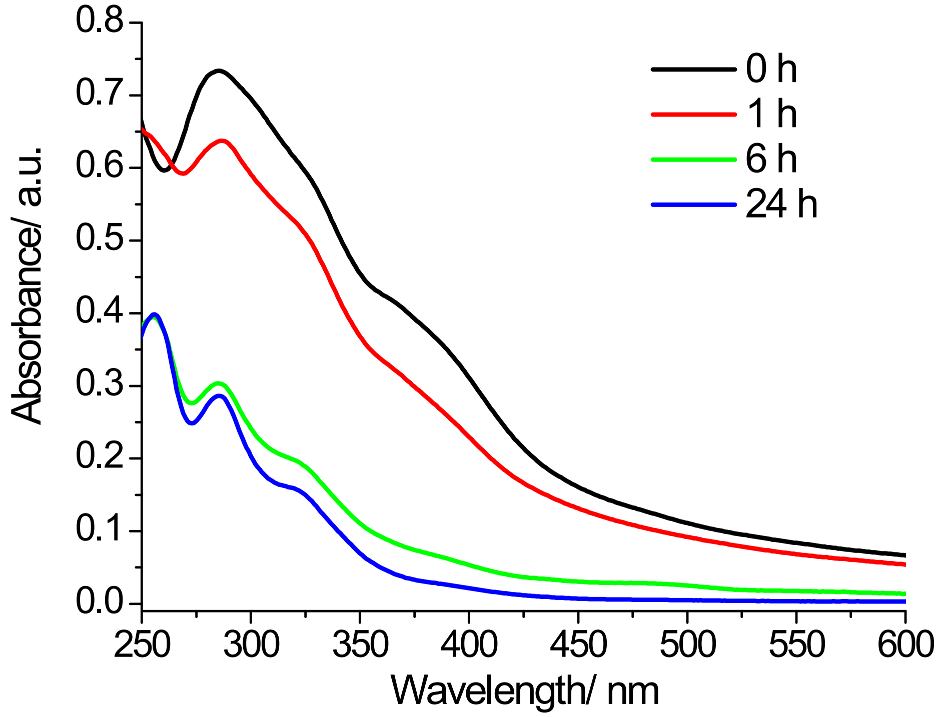

Fig. S4 UV-Vis spectrum of **1** (25 μM) in PBS:DMSO (200:1) in the presence of ascorbic acid (250 μM) over the course of 24 h at 37 °C.

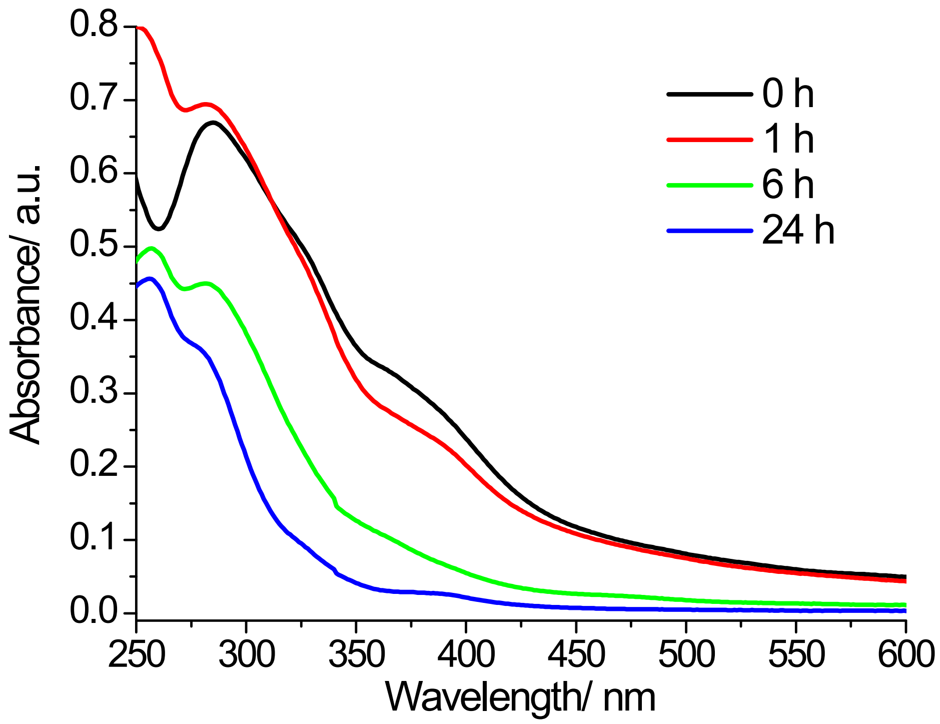

Fig. S5 UV-Vis spectrum of **1** (25 μM) in PBS:DMSO (200:1) in the presence of glutathione (250 μM) over the course of 24 h at 37 °C.

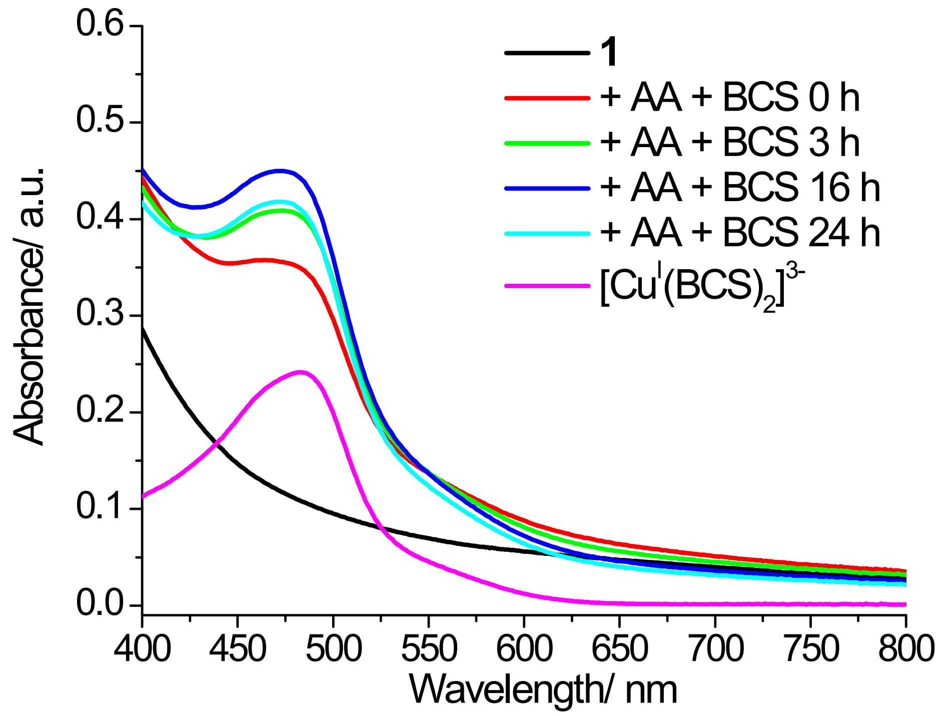

Fig. S6 UV-Vis spectrum of **1** (50 μM) in the presence of ascorbic acid (500 μM) and bathocuproine disulfonate, BCS (100 μM) in PBS:DMSO (200:1) over the course of 24 h at 37 °C.

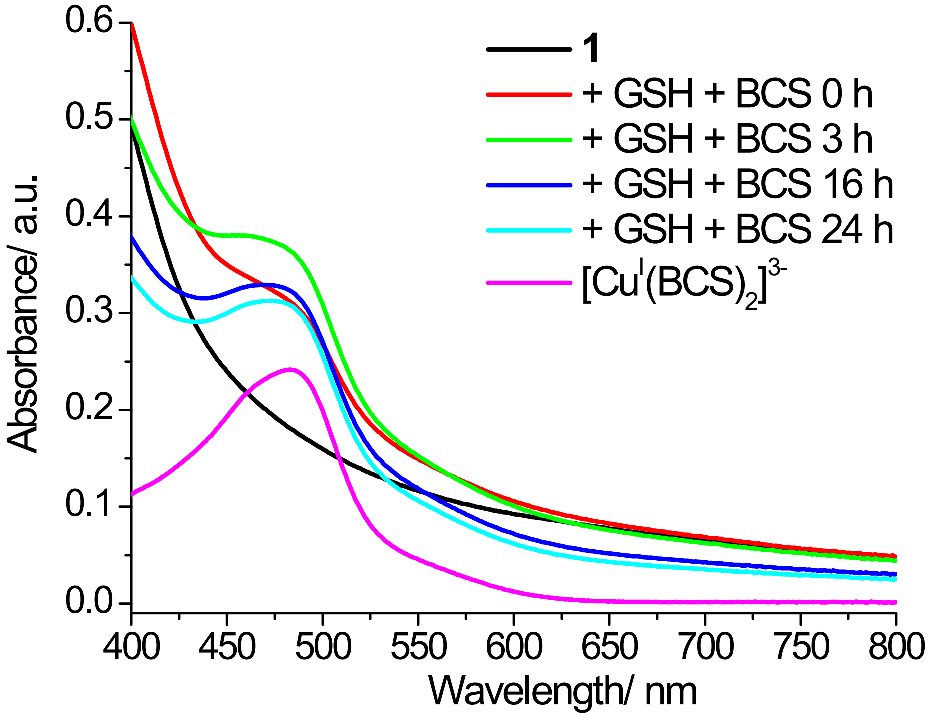

Fig. S7 UV-Vis spectrum of **1** (50 μM) in the presence of glutathione (500 μM) and bathocuproine disulfonate, BCS (100 μM) in PBS:DMSO (200:1) over the course of 24 h at 37 ^{o}C.

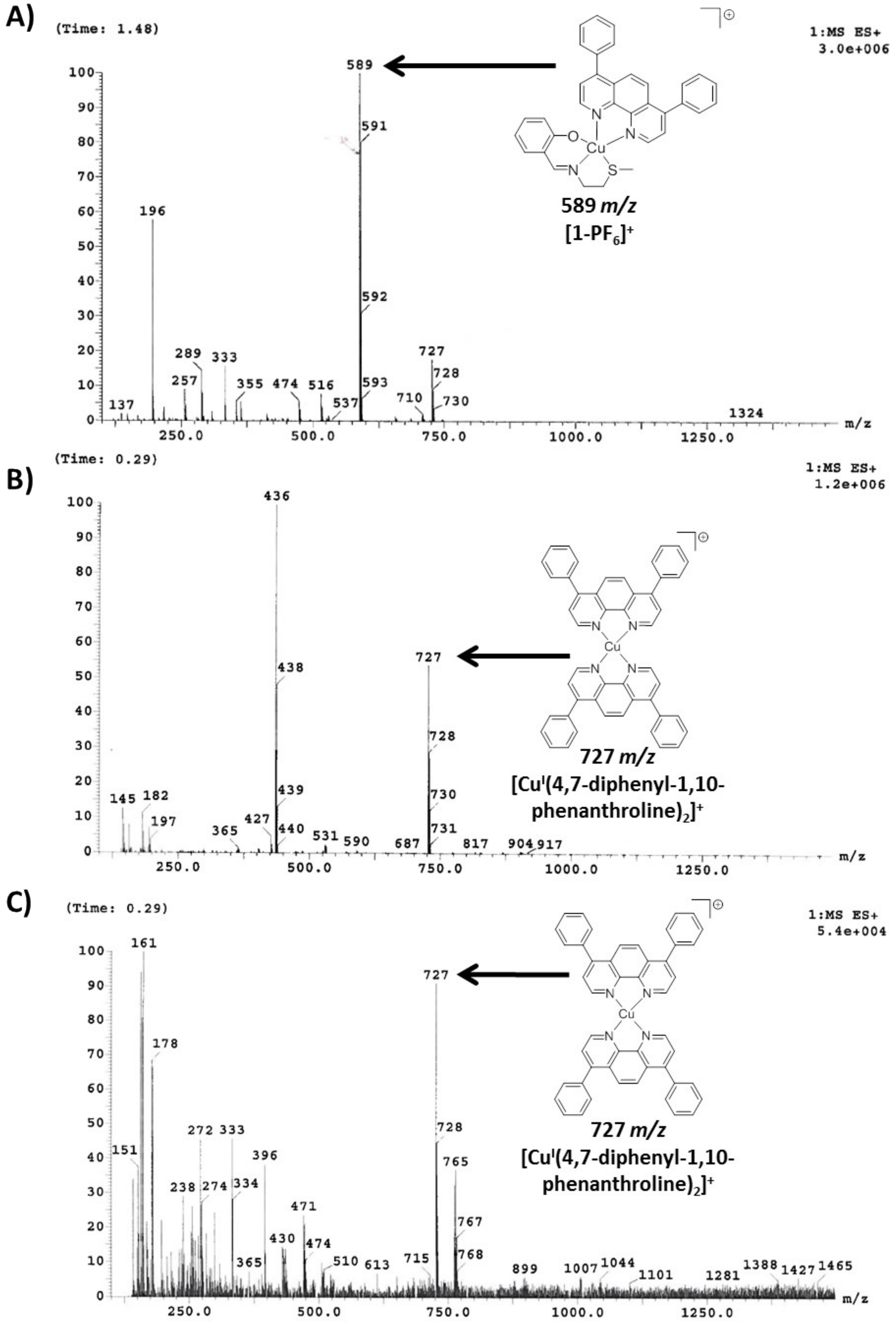

Fig. S8 ESI mass spectra (positive mode) of **1** (500 μM) in H_2O:DMSO (10:1) (A), and in the presence of ascorbic acid (5 mM) (B) or in the presence of glutathione (5 mM) (C) after incubation for 24 h at 37 ^oC.

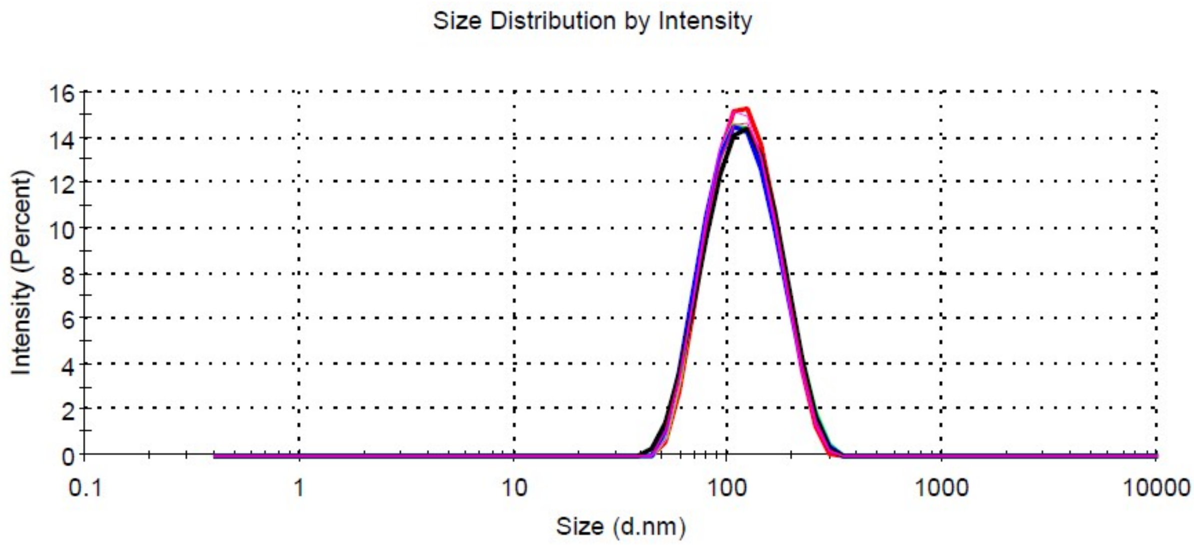

Fig. S9 Dynamic light scattering size distribution of **1 NP**[10] suspended in water. Size refers to diameter of nanoparticles in nm.

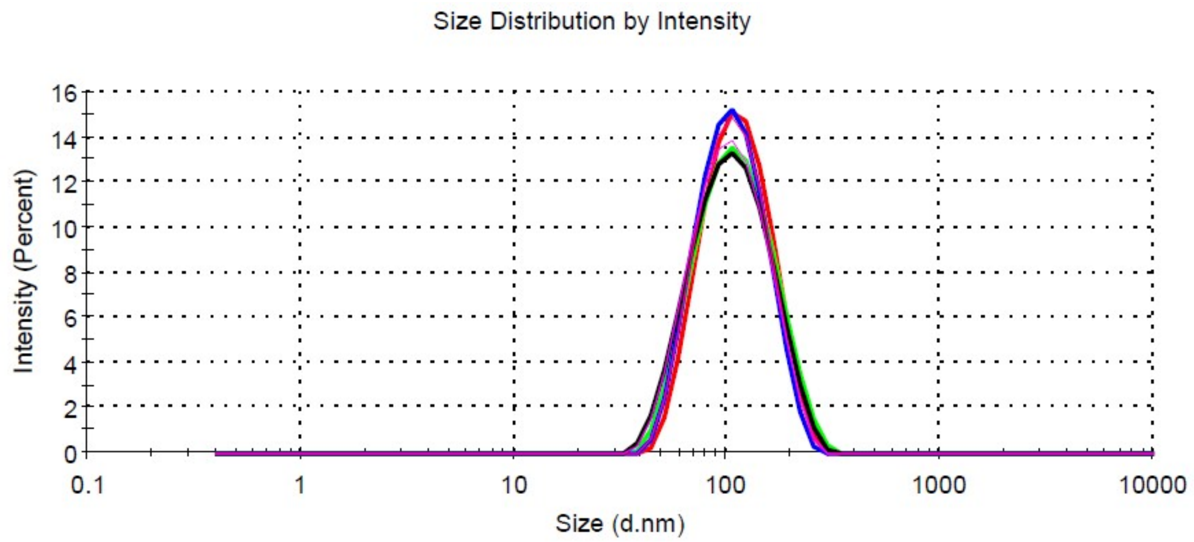

Fig. S10 Dynamic light scattering size distribution of empty PEG–PLGA nanoparticles suspended in water. Size refers to diameter of nanoparticles in nm.

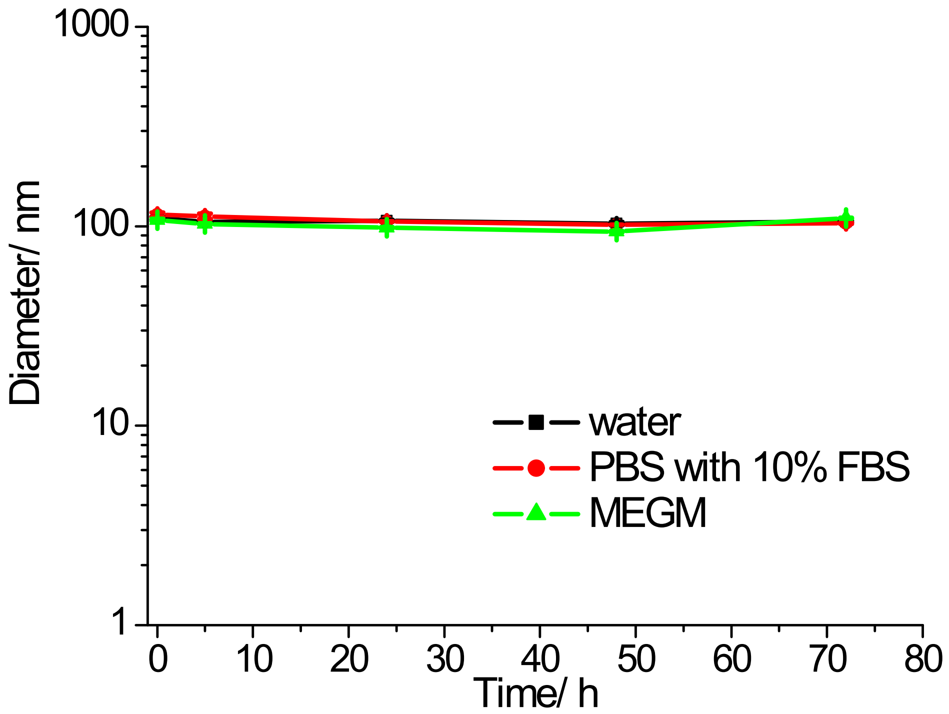

Fig. S11 Variation in **1 NP**[10] diameter upon incubation in water, PBS with 10% FBS, and mammary epithelial growth medium (MEGM) over the course of 72 h at 37 °C.

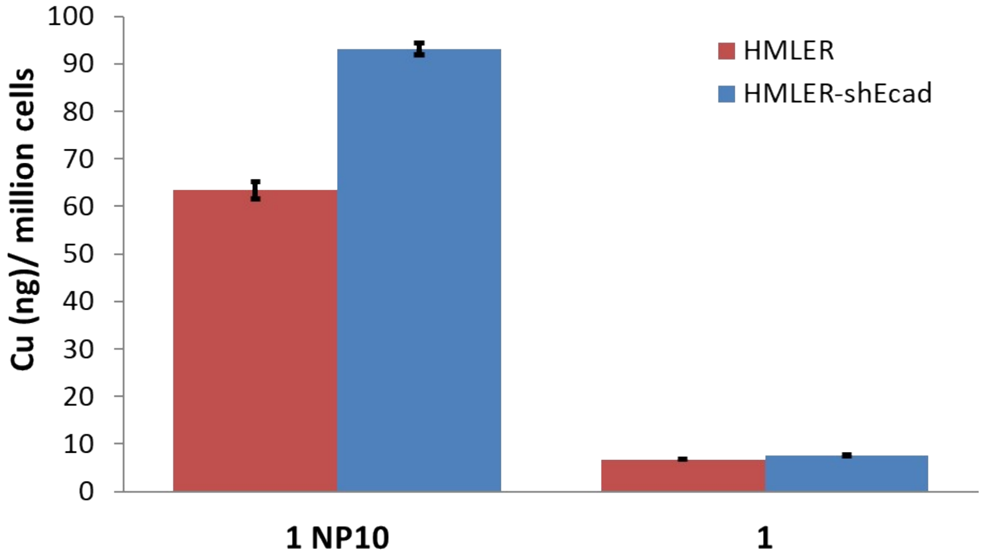

Fig. S12 Copper content in HMLER and HMLER-shEcad cells treated with **1 NP**[10] (110 nM for 24 h) or **1** (110 nM for 24 h) at 37 °C.

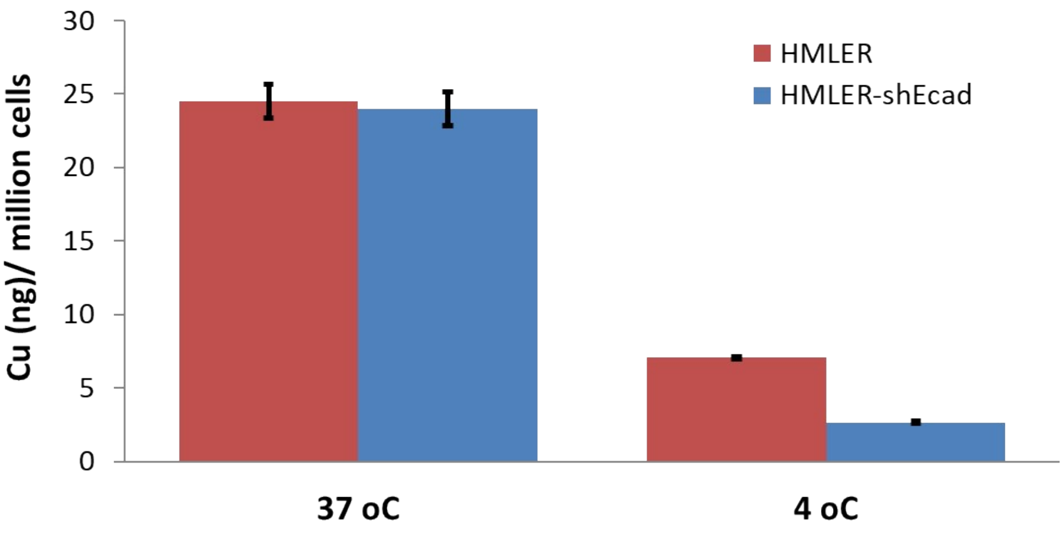

Fig. S13 Copper content in HMLER and HMLER-shEcad cells treated with **1 NP**[10] (110 nM for 4 h) at 4 °C or 37 °C.

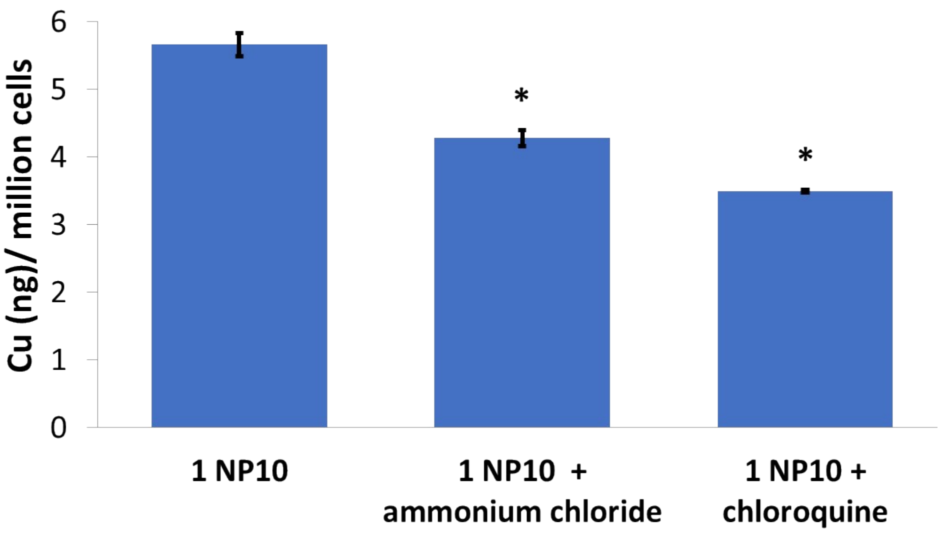

Fig. S14 Copper content in HMLER-shEcad cells treated with **1 NP**[10] only (16 nM for 24 h), and upon pre-incubation with ammonium chloride (50 mM for 2 h) or chloroquine (100 µM for 2 h) at 37 °C. Error bars represent standard deviations and Student *t test*, * = $p < 0.05$.

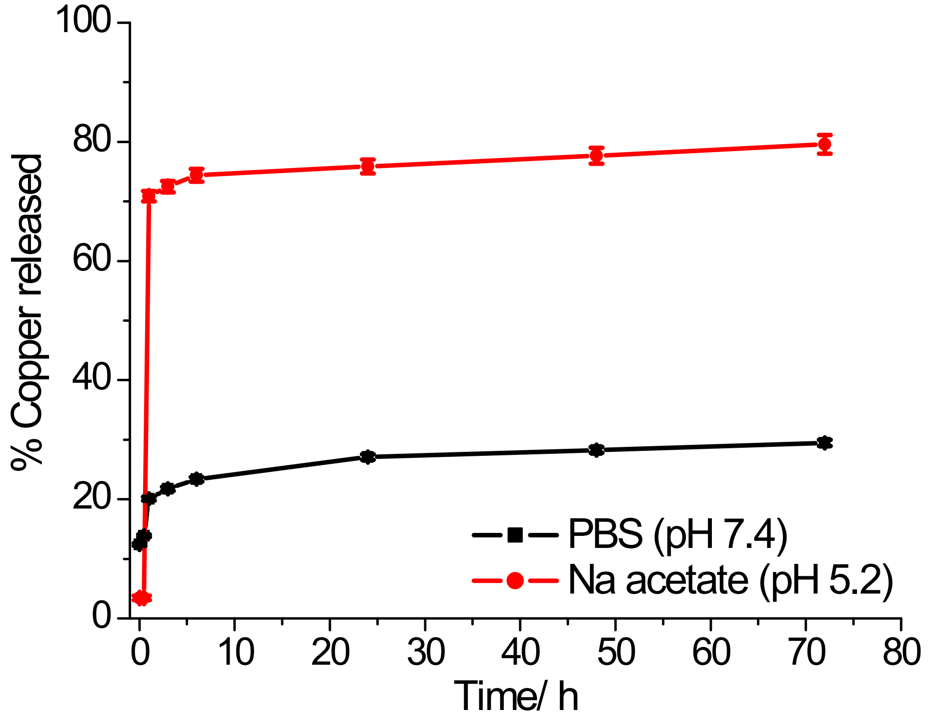

Fig. S15 The amount of copper released from **1 NP**[10] upon incubation in PBS (pH 7.4) or sodium acetate buffer (pH 5.2) over the course of 72 h at 37 °C.

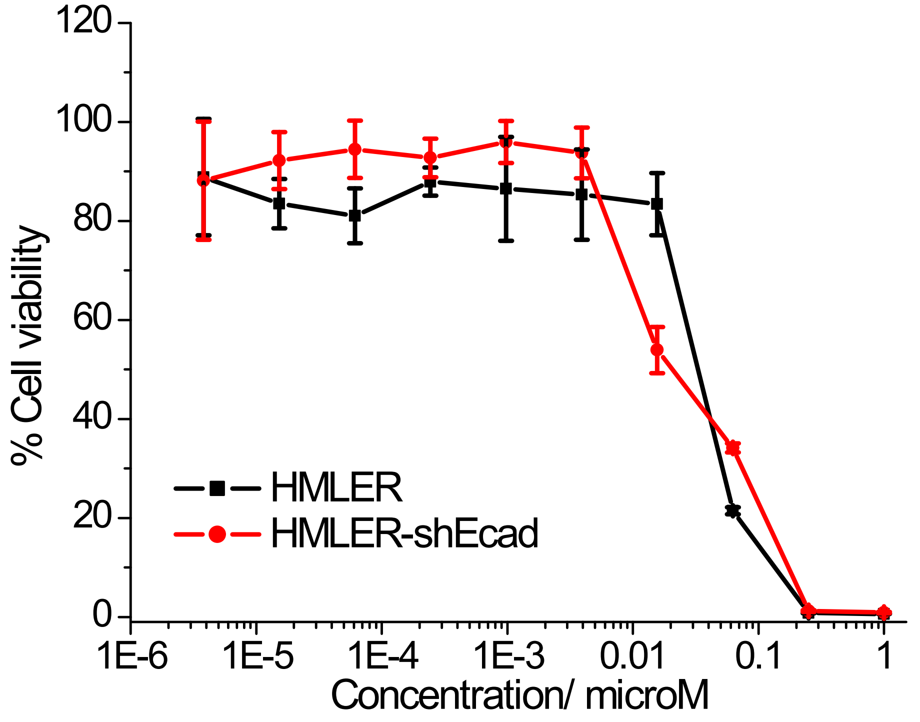

Fig. S16 Representative dose-response curves for the treatment of HMLER and HMLER-shEcad cells with **1 NP**[10].

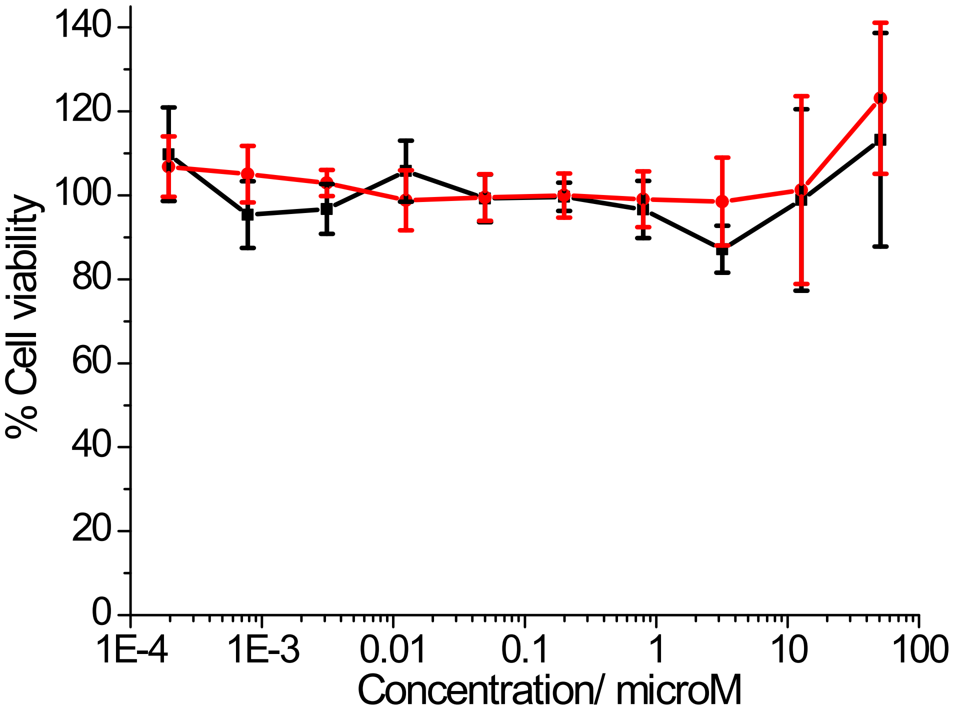

Fig. S17 Representative dose-response curves for the treatment of HMLER and HMLER-shEcad cells with empty PEG–PLGA nanoparticles.

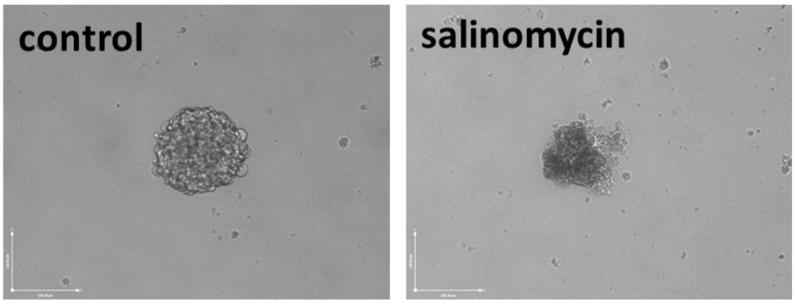

Fig. S18 Representative bright-field images (× 10) of HMLER-shEcad mammospheres in the absence and presence of salinomycin at its respective IC_{20} values for 5 days.

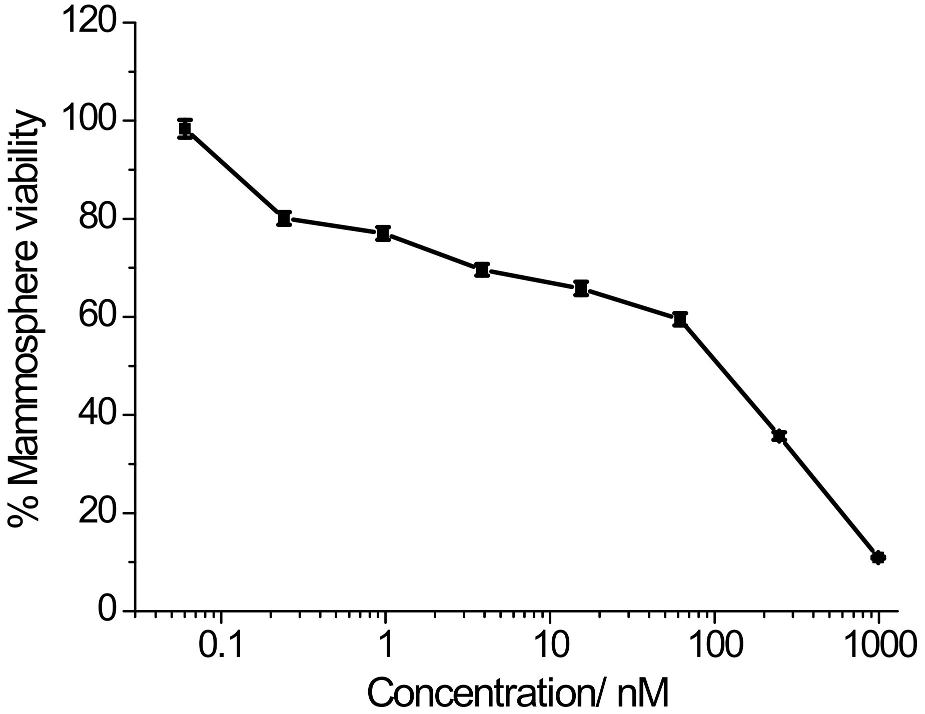

Fig. S19 Representative dose-response curve for the treatment of HMLER-shEcad mammospheres with **1 NP**[10].

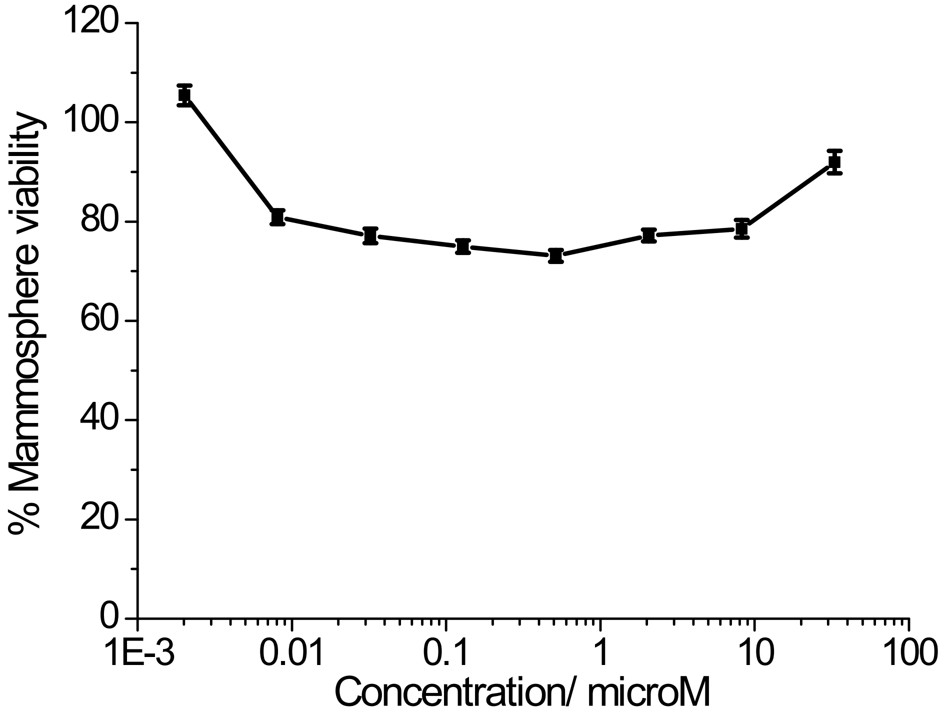

Fig. S20 Representative dose-response curve for the treatment of HMLER-shEcad mammospheres with empty PEG–PLGA nanoparticles.

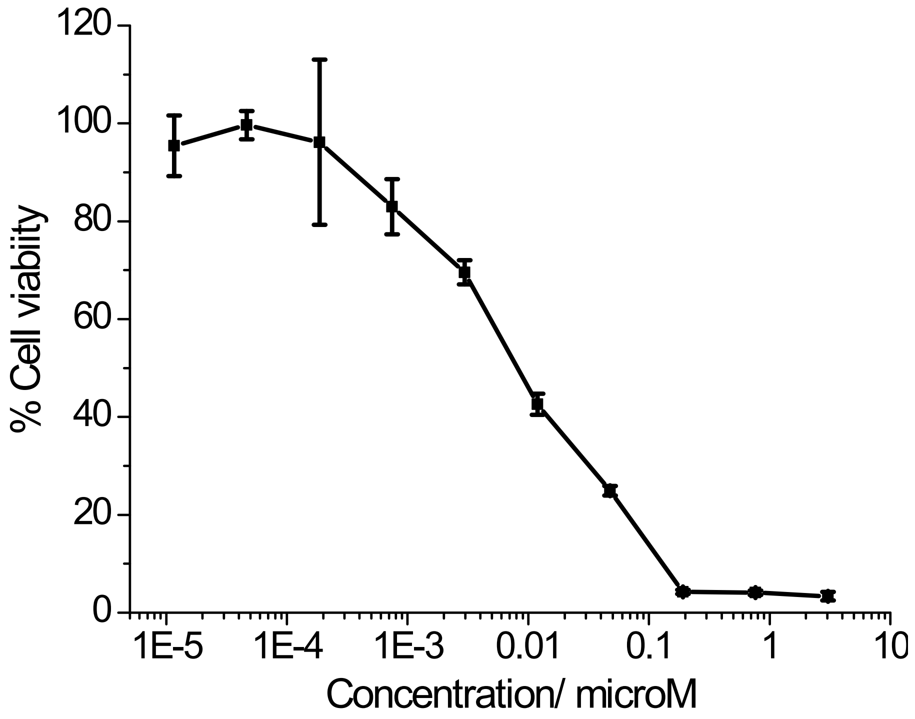

Fig. S21 Representative dose-response curves for the treatment of HMLER-shEcad cells with **1 NP**^{10} after 72 h incubation in the presence of salubrinal (10 μM).

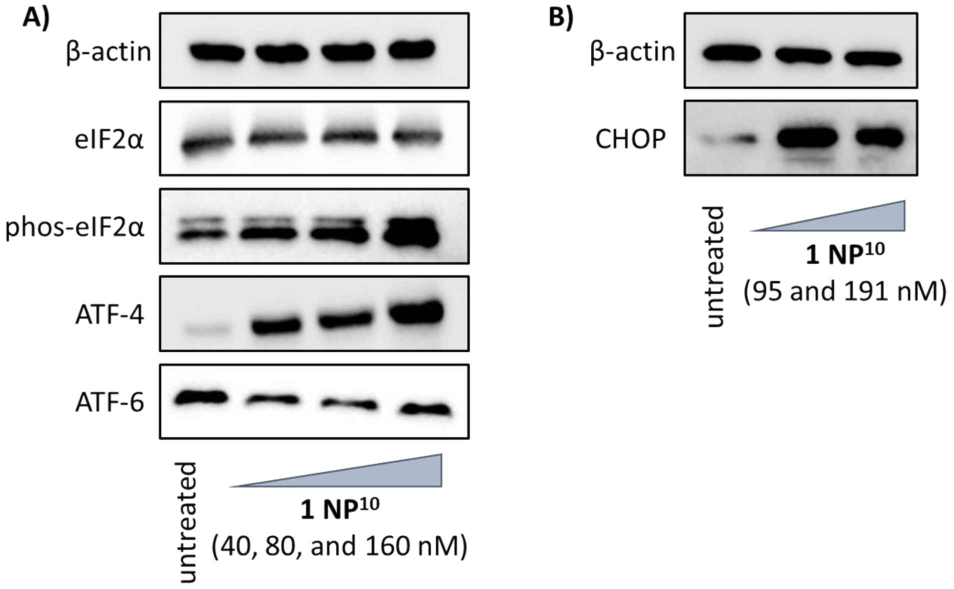

Fig. S22 Immunoblotting analysis of proteins related to the unfolded protein response (UPR). Protein expression in HMLER-shEcad cells following treatment with **1 NP**^{10} (40–191 nM) for (A) 4 h or (B) 24 h.

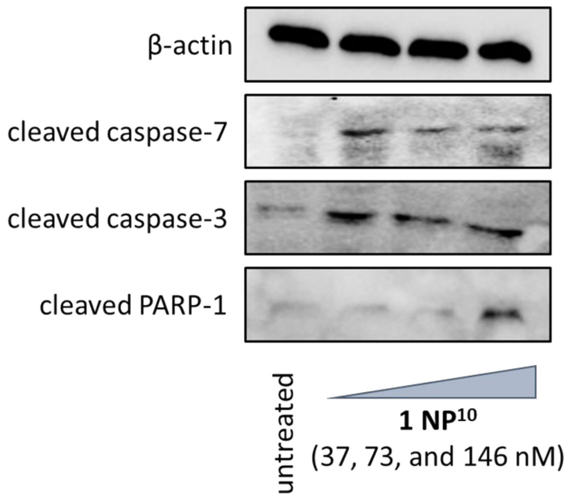

Fig. S23 Immunoblotting analysis of proteins related to apoptosis. Protein expression in HMLER-shEcad cells following treatment with **1 NP**[10] (37–146 nM for 72 h).

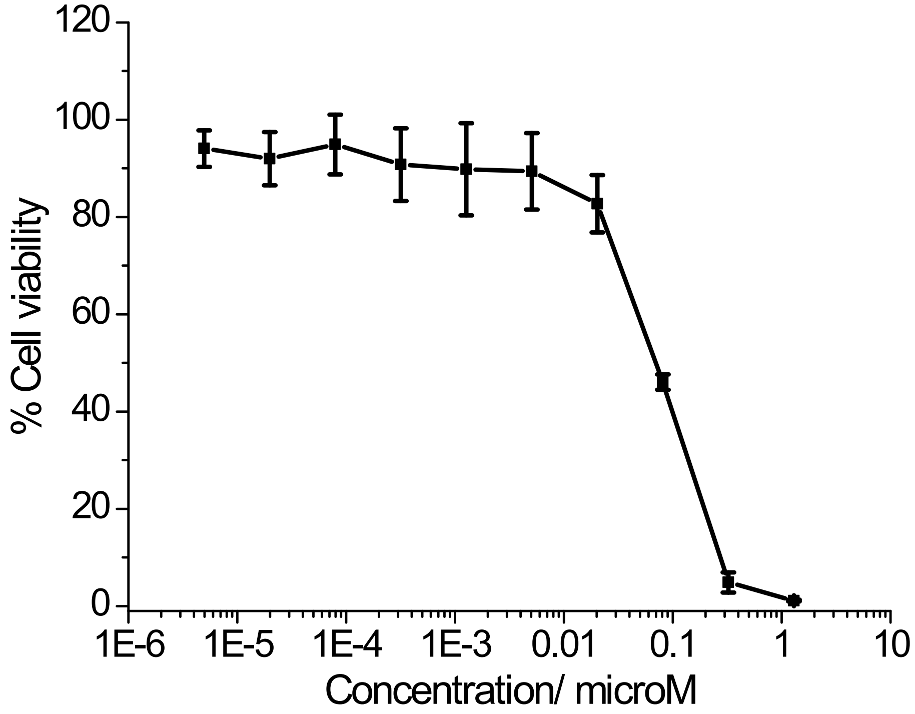

Fig. S24 Representative dose-response curves for the treatment of HMLER-shEcad cells with **1 NP**[10] after 72 h incubation in the presence of z-VAD-FMK (5 μM).

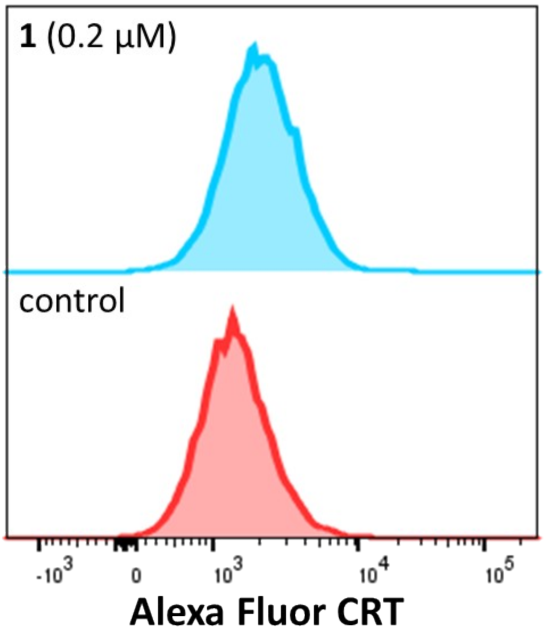

Fig. S25 Representative histograms displaying the green fluorescence emitted by anti-CRT Alexa Fluor 488 nm antibody-stained HMLER-shEcad cells untreated (red), and treated with **1** (0.2 µM for 24 h) (blue).

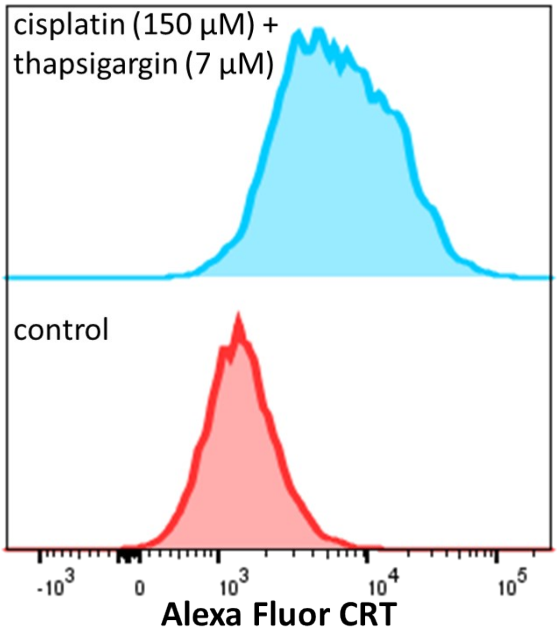

Fig. S26 Representative histograms displaying the green fluorescence emitted by anti-CRT Alexa Fluor 488 nm antibody-stained HMLER-shEcad cells untreated (red), and treated with cisplatin (150 µM for 24 h) and thapsigargin (7 µM for 24 h) (blue).

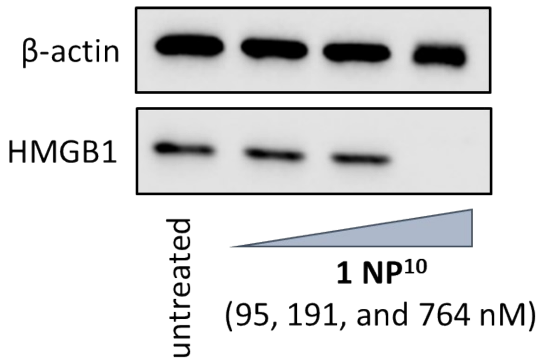

Fig. S27 Immunoblotting analysis of high mobility group box 1 (HMGB-1). Protein expression in HMLER-shEcad cells following treatment with **1 NP^{10}** (95–764 nM for 24 h).
